# An MEDT Study of the Reaction Mechanism and Selectivity of the Hetero-Diels–Alder Reaction Between 3-Methylene-2,4-Chromandione and Methyl Vinyl Ether

**DOI:** 10.3390/molecules29215109

**Published:** 2024-10-29

**Authors:** Abderrazzak Bouhaoui, Aziz Moumad, Luis R. Domingo, Latifa Bouissane

**Affiliations:** 1 Molecular Chemistry, Materials and Catalysis Laboratory, Faculty of Sciences and Technologies, Sultan Moulay Slimane University, BP 523, Beni-Mellal 23000, Morocco; abderazakbohaoui@gmail.com (A.B.); azizmoumad2@gmail.com (A.M.); 2 Department of Organic Chemistry, University of Valencia, Dr. Moliner 50, 46100 Burjassot, Valencia, Spain

**Keywords:** molecular electron density theory, hetero-Diels–Alder, ambident heterodiene, 3-methylene-2,4-chromandione, methyl vinyl ether, *pseudocyclic* selectivity, relative interacting atomic energy

## Abstract

The hetero-Diels–Alder (HDA) reaction between the ambident heterodiene 3-methylene-2,4-chromandione (MCDO) and non-symmetric methyl vinyl ether (MVE) is investigated using the molecular electron density theory (MEDT) at the B3LYP/6-311G(d,p) computational level. The aim of this study is to gain insight into its molecular mechanism and to elucidate the factors that control the selectivity found experimentally. DFT-based reactivity indices reveal that MCDO exhibits strong electrophilic characteristics, while MVE displays a strong nucleophilic character. Meanwhile, the Parr function explains the *ortho* regioselectivity of this HDA reaction. The highly polar nature of this HDA reaction, supported by the high global electron density transfer (GEDT) taking place at the transition state structures (TSs), accounts for the very low activation energy associated with the most favorable **TS-4on**. The ambident nature of MCDO allows for the formation of two constitutional isomeric cycloadducts. In the case of MVE, *pseudocyclic* selectivity is attained using a thermodynamic control. This polar HDA reaction displays an *endo* stereoselectivity and a complete *ortho* regioselectivity. A comparative relative interacting atomic energy (RIAE) analysis of the two diastereomeric structures **TS-4on** and **TS-6on** indicates a high degree of likeness, which explains the low *pseudocyclic* selectivity under kinetic control.

## 1. Introduction

Diels–Alder (DA) reactions between a diene and an ethylene derivative are one of the most studied organic reactions, enabling the construction of any six-membered carbocyclic compound [1,2,3]. Often, when the diene or the ethylene incorporates a heteroatom, the resulting reaction is called a hetero-Diels–Alder (HDA) reaction [4]. This type of reaction is a powerful tool in synthetic organic chemistry as it can be used to construct chemo-, regio-, and stereoselective heterocycles [5,6,7,8,9]. Consequently, HDA reactions provide an effective method for the synthesis of a diverse array of hetero- and polycyclic compounds [10]. The heterocycles formed are of significant biological importance and utility in a multitude of fields. This is evidenced by the fact that they constitute over half of the catalogue of known organic compounds [11].

The majority of natural compounds, including those belonging to the classes of alkaloids, vitamins, hormones, and antibiotics, contain heterocyclic moieties [12]. Coumarin and its derivatives are one of the most important classes of natural compounds widely distributed in the natural kingdom and exhibit various biological activities [13]. A number of processes for the synthesis of these heterocyclic molecules have also been developed [14] and used as precursors in many reactions such as cycloaddition [15].

**Scheme 1:**
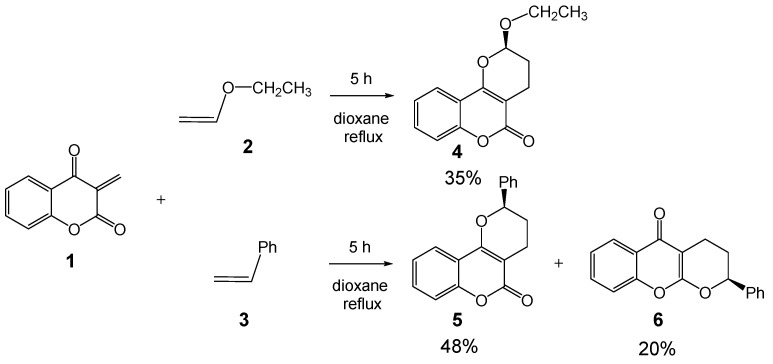
HDA reactions of MCDO **1** with olefins **2** and **3**.

In 1994, Appendino et al. reported the DA trapping of 3-methylene-2,4-chromandione (MCDO) **1** with a series of ethylene derivatives (see Scheme 1) [16]. When a non-symmetric ethylene such as ethyl vinyl ether (EVE) **2** was used, the corresponding HDA reaction took place yielding only the cycloadduct (CA) **4** with a yield of 35%; the use of styrene **3** yielded a mixture of two constitutional isomeric CAs **5** and **6** with yields of 48% and 20%, respectively. In both cases, the HDA reactions took place with total *ortho* regioselectivity [16].

**Scheme 2:**
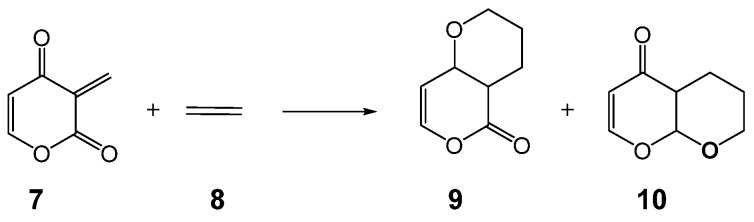
HDA reactions of the ambident compound **7** with ethylene **8**.

A theoretical study of the HDA reaction of the model ambident compound **7** with ethylene **8** using AM1 and RHF calculations was performed (see Scheme 2) [16]. The activation enthalpy for the formation of the constitutional isomeric CA **10** was 4.10 kcal·mol^−1^ higher in energy than that associated with the formation of CA **9**, 23.27 kcal·mol^−1^, in agreement with the selectivity experimentally observed in the HDA reaction with isoprene **11**. In order to explain the suggested chemo- and regioselectivity of these HDA reactions, an AM1 analysis of the LUMO coefficients of MCDO **1** and the HOMO coefficients of the non-symmetric ethylenes as ethyl vinyl ether **2** was carried out.

Based on many theoretical studies of experimental DA reactions, a very good correlation between the activation energies of the DA reactions and the global electron density transfer [17] (GEDT) taking place at the transition state structures (TSs) was recognized in 2009. Thus, the general mechanism of polar Diels–Alder (P-DA) reactions was established [18], which proves that P-DA reactions are controlled by the nucleophilic and electrophilic interactions taking place at the TSs. In this context, the analysis of DFT-based reactivity indices [19,20] has become a powerful tool to investigate polar cycloaddition reactions [21].

In 2016, Domingo proposed the molecular electron density theory [22] (MEDT) to study chemical organic reactivity. This theory states that the energy cost associated with the reorganization of the molecular electron density along a reaction path is what determines the chemical reactivity. In MEDT studies, a wide range of quantum chemical tools are used that allow for the analysis of density functions, such as the DFT-based reactivity indices [19,20], the electron localization function [23] (ELF), the bonding evolution theory [24] (BET), the quantum theory of atoms in molecules [25,26] (QTAIM), and the non-covalent interaction [27] (NCI). Very recently, an energy decomposition analysis based on interacting quantum atoms [28] (IQAs), relative interacting atomic energy [29] (RIAE), has been proposed to analyze the factors controlling activation energies, i.e., the relative energies of the TSs in respect to the reference species, i.e., reagents or molecular complexes. By applying an RIAE analysis in three studies of polar DA reactions, it has been proven that the GEDT taking place from the nucleophile to the electrophile is the main electronic factor responsible for the reduction in the activation energies in polar reactions [30].

**Scheme 3:**
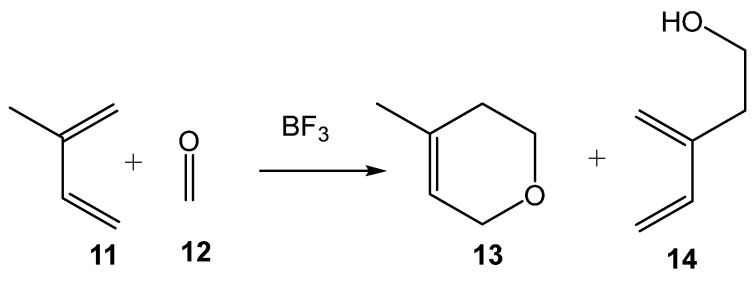
Competitive HDA and Alder-ene reactions in the Lewis acid catalyzed reaction between isoprene **11** and formaldehyde **12**.

In 2018, Domingo et al. studied within the MEDT the Lewis acid catalyzed HDA reaction between isoprene **11** and formaldehyde **12** [31]. Together with the expected CA **13**, the reaction path yielding the constitutional isomeric Alder-ene adduct **14** was characterized (see Scheme 3). This MEDT study allowed for the establishment of the similarity of the two diastereomeric TSs associated with the formation of the first C–C single bond along the two competitive reaction paths. In this work, the term *pseudocyclic* selectivity was suggested to denote the selective formation of a constitutional isomer through diastereomeric *pseudocyclic* TSs [31].

In the present work, an MEDT study of the HDA reaction of MCDO **1** with methyl vinyl ether **15** (MVE), as a model of the experimental ethyl vinyl ether **2** used by Appendino et al. [16] (see Scheme 1), is carried out in order to establish the molecular mechanism of these HDA reactions as well as the origin of the selectivity experimentally observed.

## 2. Results and Discussion

This MEDT study is divided into five parts: (i) first, an ELF topological analysis of MCDO **1** and MVE **15** in their ground state was carried out; (ii) next, an analysis of the DFT-based reactivity indices was performed to characterize the reactivity of these species participating on polar HDA reactions, as well as to characterize the specific active sites of each reagent; (iii) then, the competitive reaction paths associated with the HDA reaction between MCDO **1** and MVE **15** were studied; (iv) after that, the electronic structure of the more favorable TSs for the formation of CA **4** and its possible constitutional isomeric CA was characterized by topological ELF and QTAIM analyses; (v) and finally, an RIAE analysis of the HDA reactions of MCDO **1** with MVE **15** and with ethylene **8** was performed in order to characterize the electronic factor controlling the activation energies of the HDA reaction.

### 2.1. Topological Analysis of the ELF of MCDO ***1*** and MVE ***15***

**Figure 1:**
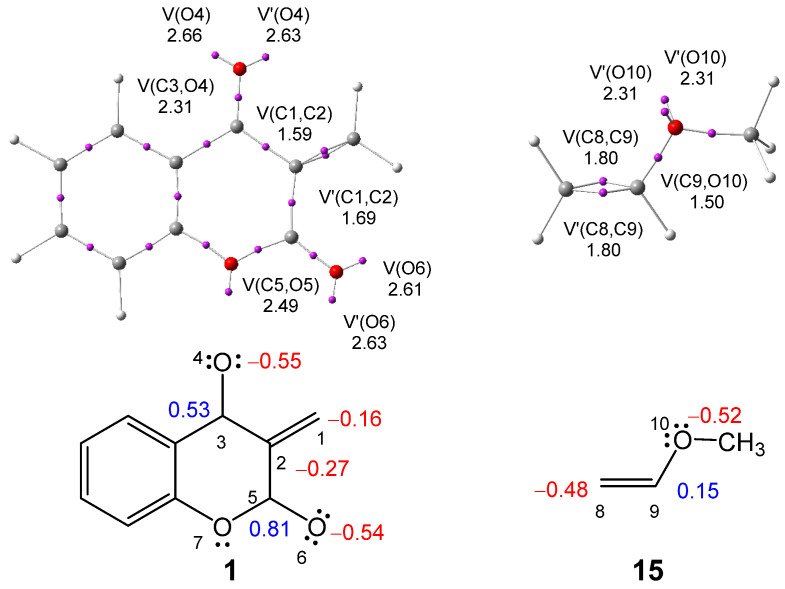
B3LYP/6-311G(d,p) ELF basin attractor positions, populations of the most relevant valence basins, and natural atomic charges of MCDO **1** and MVE **15**. Valence basin populations and natural atomic charges are given as average number of electrons, e. Negative charges are shown in red, and positive charges are shown in blue.

The topological analysis of the ELF [23] is a valuable method to study the distribution of electron density in a molecule [32], thereby facilitating the prediction of its chemical reactivity. Thus, an ELF topological analysis of the electronic structures of MCDO **1** and MVE **15** was first performed. ELF basin attractor positions, together with the most relevant valence basin populations, are shown in Figure 1.

The ELF of MCDO **1** shows the presence of two disynaptic basins, V(C1,C2) and V′(C1,C2), integrating a total of 3.28 e, associated with a depopulated C1–C2 double bond; one V(C3,O4) disynaptic basin, integrating 2.31 e, characterizing the C3–O4 bonding region as a single bond; two monosynaptic basins, V(O4) and V′(O4), integrating a total population of 5.92 e, associated with the non-bonding electron density at the O4 oxygen atom; one V(C5,O6) disynaptic basin, integrating 2.49 e, characterizing the C5–O6 bonding region as a single bond; and two monosynaptic basins, V(O6) and V′(O6), integrating a total of 5.24 e, associated with the non-bonding electron density at the O6 oxygen atom. On the other hand, the ELF of MVE **15** reveals the presence of two disynaptic basins, V(C8,C9) and V′(C8,C9), integrating a total of 3.60 e, associated with a depopulated C8–C9 double bond, and one V(C9,O10) disynaptic basin integrating 1.50 e associated with a depopulated C9–O10 single bond.

The Lewis-like structures of MCDO **1** and MVE **15**, together with their natural charges [33,34], are shown in Figure 1. The natural charges of MCDO **1** indicate that the olefinic C1 and C2 carbons are negatively charged by −0.16 and −0.27 e, respectively, while carbonyl C3 and C5 carbons are strongly positively charged by 0.53 and 0.81 e due to the strong polarization of the carbonyl C–O bonding regions under the effect of the electronegative O4 and O6 oxygen atoms, which are negatively charged by −0.55 e. For MVE **15**, the C8 carbon is negatively charged by −0.48 e while the C9 carbon is positively charged by 0.15 e, indicating the strong charge polarization of the C8–C9 single bond.

### 2.2. Analysis of the DFT-Based Reactivity Indices in the Ground State of the Reagents

Analyses of DFT-based reactivity indices [19,20] are powerful tools to understand the reactivity in polar cycloaddition reactions. The reactivity indices were calculated at the B3LYP/6-31G(d) computational level since it was used to establish the electrophilicity and nucleophilicity scales [20]. The global reactivity indices, namely, the electronic chemical potential μ, chemical hardness η, global electrophilicity ω, and global nucleophilicity *N* of MCDO **1** and MVE **15** are given in Table 1.

The electronic chemical potential [35] μ of MCDO **1**, μ *=* −4.88 eV, is positioned below that of MVE **15**, μ *=* −2. 34 eV. The high energy difference between these values indicates that this HDA reaction will have a high polar character, with the GEDT [17] taking place from MVE **15** towards MCDO **1**.

On one hand, MCDO **1** has an electrophilicity ω index [36] of 2.87 eV, being classified as a strong electrophile within the electrophilicity scale [20], and a nucleophilicity *N* index [37] of 2.16 eV, being classified as a moderate nucleophile within the nucleophilicity scale [20]. On the other hand, MVE **15** has an electrophilicity ω index of 0.42 eV, being classified as a marginal electrophile, and a nucleophilicity *N* index of 3.20 eV, being classified as a strong nucleophile.

The strong electrophilic character of MCDO **1** combined with the strong nucleophilic character of MVE **15** means that the corresponding HDA reaction will have a remarkable polar character, being characterized as reverse electron density flux (REDF) [38].

Ethylene **8** characterized with an ω = 0.73 eV and an *N* = 1.86 eV is classified as a marginal electrophile and a marginal nucleophile. Consequently, it is expected that ethylene **8** will have a low tendency to participate in polar cycloaddition reactions.

**Figure 2:**
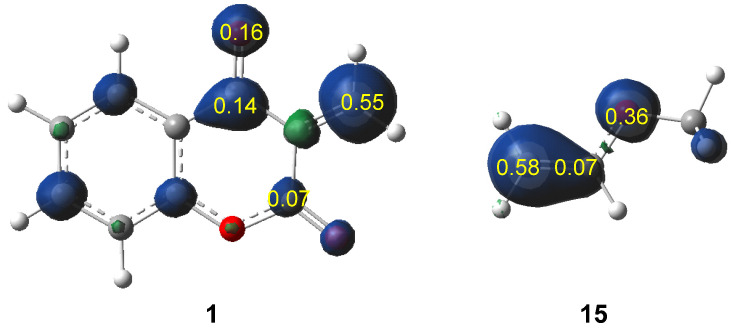
3D representations of the Mulliken atomic spin densities of the radical anion of MCDO **1** and of the radical cation of MVE **15**, together with the electrophilic $P_{k}^{+}$ Parr functions of MCDO **1** and the nucleophilic $P_{k}^{-}\;\mathrm{Parr}$ functions of MVE **15**.

In a polar cycloaddition reaction involving non-symmetric species, the most favorable reaction path takes place at the two-center interaction between the more electrophilic center and the more nucleophilic center of the two interacting reagents. Numerous studies have indicated that the analysis of Parr functions [39], derived from the excess spin electron density collected by the GEDT, is one of the most effective means of analyzing local reactivity in polar and ionic processes. Consequently, the electrophilic Parr $P_{\kappa }^{+}$ functions of MCDO **1** and the nucleophilic $P_{\kappa }^{-}$ Parr functions of MVE **15** were studied (see Figure 2).

The analysis of the electrophilic $P_{\kappa }^{+}$ Parr functions of the electrophilic MCDO **1** shows that the C1 carbon, $P_{\kappa }^{+}$ = 0.55, is the most electrophilic center in this molecule. It is noticed that 55% of the electrophilicity of this molecule is concentrated in this carbon. Similarly, the analysis of the nucleophilic $P_{\kappa }^{-}$ Parr functions of MVE **15** indicates that the C8 carbon, $P_{\kappa }^{-}$ = 0.58, is the most nucleophilic center in this molecule.

Therefore, the analysis of the Parr functions predicts that the most likely electrophilic/nucleophilic interaction along the nucleophilic attack of MVE **15** on MCDO **1** will be between the C8 of the former and the C1 carbon of the latter, providing an explanation for the *ortho* regioselectivity observed in the DA reactions [40].

It is worth emphasizing that the most electrophilic center of MCDO **1** is the C1 carbon, which is negatively charged, −0.16 e, while the two carbonyl C3 and C4 carbons are positively charged (see Figure 1). This finding supports the Domingo’s proposal made in 2014 where the local electrophilic/nucleophilic behaviors of organic molecules are not simply caused by charges [41].

### 2.3. Analysis of Competitive Reaction Paths Associated with the HDA Reaction of MCDO ***1*** with MVE ***15***

**Scheme 4:**
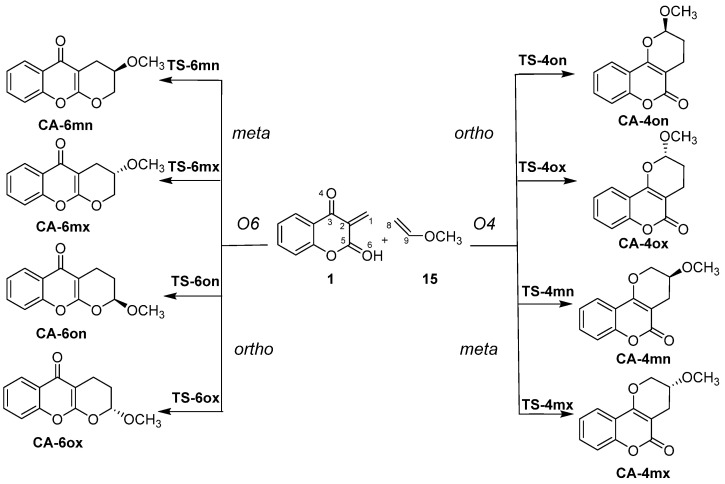
The eight competitive reaction paths associated with the HDA reaction of MCDO **1** with MVE **15**.

Due to the non-symmetric character of the two reagents and the presence of two heterodiene frameworks in MCDO **1**, eight competitive reaction paths are feasible along the nucleophilic attack of MVE **15** on the C1 carbon of MCDO **1** (see Scheme 4). They are related to the *pseudocyclic* selectivity [31] associated with the participation of the carbonyl O4 or O6 oxygen atoms of the heterodiene, named as *O4* and *O6*, respectively; the regioisomeric approach of the C8–C9 double bond of MVE **15** to the C1–C2 double bond of MCDO **1**, named as *ortho* and *meta*; and the stereoisomeric approach of the ether O19 oxygen atom of MVE **15** with respect to the heterodiene system, named as *endo* and *exo*. The eight competitive reaction paths were analyzed (see Scheme 4). The analysis of the stationary points located along the eight competitive reaction paths indicates that this HDA reaction proceeds according to a non-concerted one-step mechanism. Table 2 shows the B3LYP/6-3111G(d,p) relative electronic energies in the gas phase and in dioxane. Table S1 in the Supplementary Materials presents the corresponding total electronic energies.

Some appealing conclusions can be obtained from the gas-phase relative energies given in Table 2: (i) the activation energies associated with the eight competitive reaction paths vary from 4.21 kcal·mol^−1^ (**TS-4on**) to 25.69 kcal·mol^−1^ (**TS-6mx**), while the exothermic characteristics of these reaction paths vary from −18.46 kcal·mol^−1^ (**CA-6mx**) to −35.39 kcal·mol^−1^ (**CA-4ox**); (ii) the activation energy associated to this HDA reaction is 13.05 kcal·mol^−1^ lower in energy than that of the HDA reaction of MCDO **1** with ethylene **8** as a consequence of the strong nucleophilic character of MVE **15** (see Scheme S1 in the Supplementary Materials); (iii) the most favorable **TS-4on** is associated with the *orto/endo* approach of MVE **15** to the C1–C2–C3–O4 heterodiene system MCDO **1**; (iv) this HDA reaction is *endo* stereoselective as **TS-4ox** is located 0.67 kcal·mol^−1^ above **TS-4on**; (v) this HDA reaction has a low *pseudocyclic* selectivity as **TS-6on** is located only 0.05 kcal·mol^−1^ above **TS-4on**; (vi) this HDA reaction is completely *ortho* regioselective as **TS-4mn** is located 15.55 kcal·mol^−1^ above **TS-4on**; and (vii) the formation of **CA-4on** along the most favorable reaction path is strongly exothermic by −34.12 kcal·mol^−1^. Consequently, the formation of **CA-4on** can be considered irreversible (see later). The inclusion of the solvent effects of dioxane does not produce remarkable changes in the gas-phase energies (see Table 2).

The most favorable TSs responsible for the *pseudocyclic* selectivity found in these HDA reactions, **TS-4on** and **TS-6on**, were optimized using the experimental EVE **2**. The gas-phase B3LYP/6-311G(d,p) total and relative energies are given in Table S2 in the Supplementary Materials. The substitution of the methyl group present in MVE **15** by the ethyl group present in EVE **2** does not modify either the activation energy, 3.99 kcal·mol^−1^, or the poor *pseudocyclic* selectivity under kinetic control (ΔΔE = 0.02 kcal·mol^−1^).

**Figure 3:**
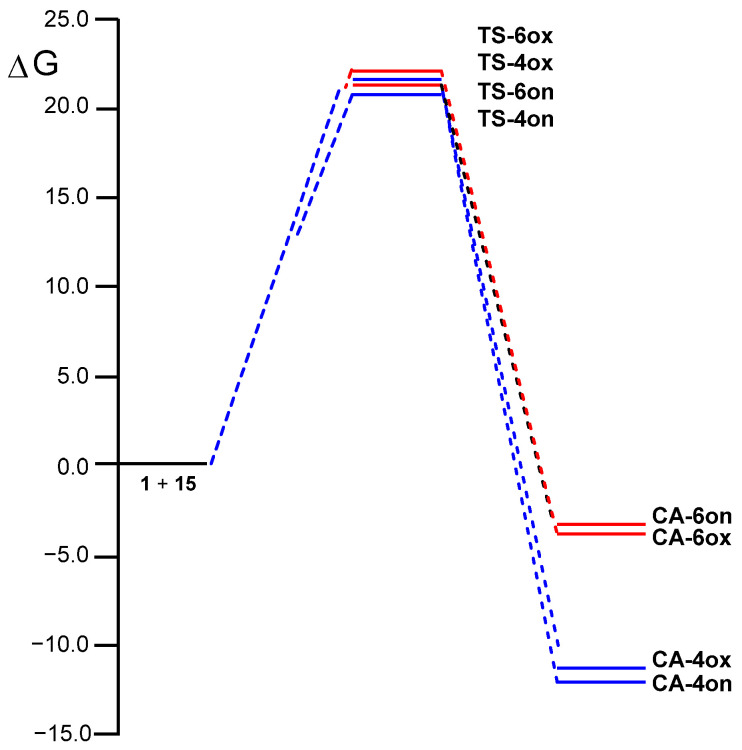
B3LYP/6-311G(d,p) Gibbs free energy profiles, ΔG in kcal·mol^−1^, computed at 101.1 °C in dioxane for the more favorable regioisomeric *ortho* reaction paths associated with HDA reaction of MCDO **1** with MVE **15**.

The relative Gibbs free energies of the stationary points associated with the more favorable regioisomeric *ortho* reaction paths associated with the HDA reaction of MCDO **1** with MVE **15** were computed at 101.1 °C in dioxane. The high activation energy of the *meta* TSs, which was more than 15.55 (**TS-4mn**) kcal·mol^−1^, discards the thermodynamic study of these non-competitive reaction paths. The thermodynamic data are given in Table S3 in the Supplementary Materials, while the corresponding Gibbs free energy profiles are given in Figure 3.

The inclusion of the thermal corrections, the entropies, and the temperature increases the activation Gibbs free energies of the most favorable reaction path via **TS-4on** to 20.93 kcal·mol^−1^. The reaction is *endo* stereoselective as **TS-4ox** is 0.85 kcal·mol^−1^ higher in Gibbs free energy than **TS-4on** and very low *O*6 *pseudocyclic* selective as **TS-6ox** is only 0.01 kcal·mol^−1^ higher in Gibbs free energy than **TS-4on**. Interestingly, at this computational level, while the *O4* reaction paths are exergonic by more than 11.4 kcal·mol^−1^, the *O6* reaction paths are exergonic by only 3.7 kcal·mol^−1^. Consequently, in dioxane under reflux conditions, the *O6/ortho/endo* reaction path can be reversible, yielding the thermodynamically more stable *O4/ortho/endo* **CA-4on** (see **8** in Scheme 1).

**Figure 4:**
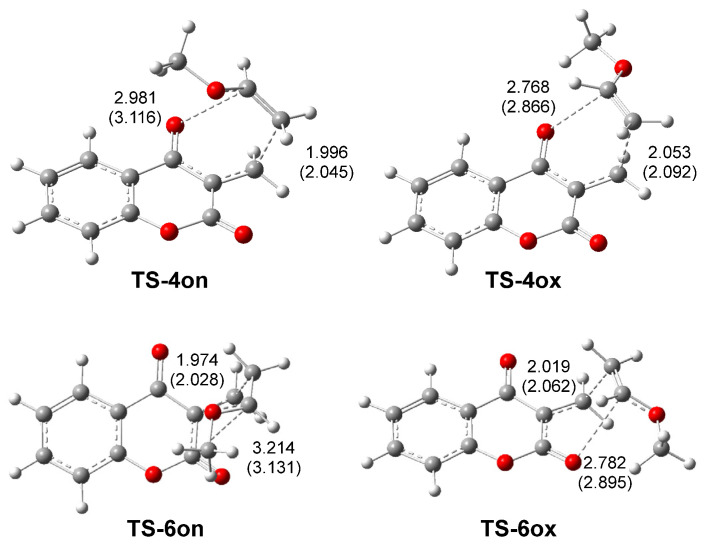
B3LYP/6-311G(d,p) geometries of the most favorable regioisomeric *ortho* TSs associated with HDA reaction of MCDO **1** with MVE **15**. Values in dioxane are given in parenthesis. Distances are given in angstroms Å.

The geometries of the most favorable regioisomeric *ortho* TSs associated with the HDA reaction of MCDO **1** with MVE **15** are given in Figure 4, while the geometries of the regioisomeric *meta* TSs are given in Figure S3 in the Supplementary Materials. Regarding the four TSs, the distance between the two interacting carbons involving the most electrophilic C1 carbon of MCDO **1** and the most nucleophilic C8 carbon of MVE **15** is found to be in the narrow range of 1.97–2.05 Å, while the distances between the C9 carbon of MVE **15** and the O4 and O6 oxygen atoms of MCDO **1** are found to be between 2.8 and 3.2 Å. These geometrical parameters indicate that this HDA reaction takes place through very high asynchronous TSs. The IRC analysis of the most favorable **TS-4on** indicates that this HDA reaction takes place through a non-concerted *one-step two-stage* mechanism [42] in which the formation of the second O4–C9 single bond begins when the formation of C1–C8 is practically completed. The addition of dioxane as a solvent does not produce remarkable changes in the geometries (see Figure 4). In fact, in the presence of dioxane, the TSs are slightly more delayed.

**Figure 5:**
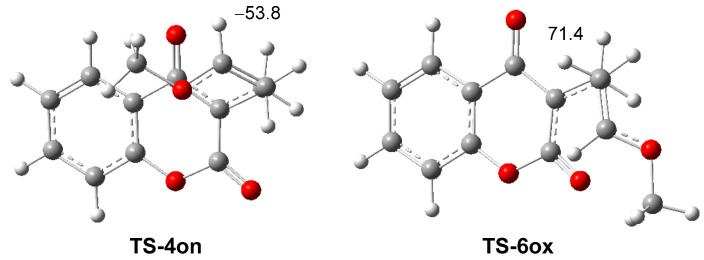
View of the diastereomeric **TS-4on** and **TS-6ox** along the axis formed by the interacting C1 and C8 carbons. The C2–C1–C8–C9 dihedral angles are given in degrees.

Figure 5 displays a representation of the stereoisomeric **TS-4on** and **TS-6ox** along the axis formed by the C1 and C8 interacting carbons. Similar to the *endo* and *exo* TSs, these TSs have a diastereomeric relationship. After passing these stereoisometic TSs, and once the formation of the first new C1–C8 single bond is fully completed, the formation of the second O4–C9 or C6–C19 single bond occurs at the end of the corresponding reaction path, permitting the formation of the constitutional isomers **CA-4on** and **CA-6ox**.

The GEDT analysis [17] at the TSs involved in this HDA reaction allows us to quantify the polar character of this reaction [18]. In polar reactions, the GEDT happening at the TSs, which fluxes from the neutrophilic towards the electrophilic species, is the main factor responsible for the stabilization of the TSs [30]. GEDT values lower than 0.05 e correspond to non-polar processes, while values higher than 0.20 e correspond to polar processes. The GEDT values at the two more favorable *ortho/endo* TSs are 0.38 e at **TS-4on** and 0.39 e at **TS-6on**. These uppermost values illustrate the high polar character of this HDA reaction and consequently the low activation energies associated with these TSs.

The flux of the electron density at these polar TSs, which goes from the strong nucleophilic MVE **15** towards the strong electrophilic MCDO **1**, classifies this HDA reaction as REDF [38], which is in complete agreement with the previous analysis of the DFT-based reactivity indices.

### 2.4. ELF and QTAIM Analyses of the Electronic Structure of the Stereoisomeric ***TS-4on*** and ***TS-6on***

**Figure 6:**
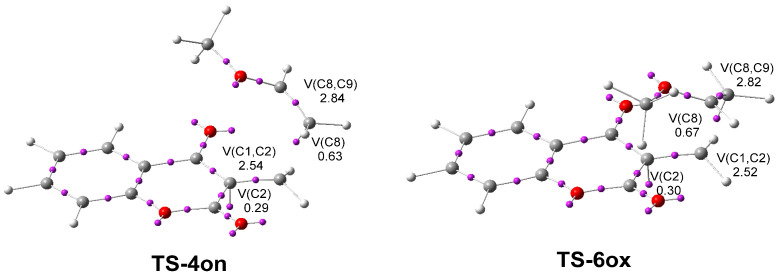
ELF basin attractor positions and populations of the most relevant valence basins of **TS-4on** and **TS-6on**. Valence basin populations are given in average number of electrons, e.

With the aim of understanding the similitude of the diastereomeric *ortho/endo* **TS-4on** and **TS-6on**, an ELF [23] and QTAIM [25,26] topological analysis of the two TSs was performed. The ELF basin attractor positions, together with the most relevant valence basin populations at the two TSs, are shown in Figure 6.

The analysis of the ELF in both TSs shows there is a great degree of similarity between them. In fact, they show the presence of two monosynaptic basins, V(C2) and V(C8), integrating ca. 0.29 and 0.65 e, respectively; one V(C1,C2) disynaptic basin, integrating ca. 2.53 e; and one V(C8,C9) disynaptic basin, integrating ca. 2.83 e. The two V(C2) and V(C8) monosynaptic basins are associated with the two C2 and C8 *pseudoradical* centers [17] created along the reaction paths. The C8 *pseudoradical* center is required for the subsequent creation of the first new C1–C2 single bond in a further step of the reaction path [17]. The electron density of these C2 and C8 *pseudoradical* centers comes from the depopulation of the C1–C2 and C8–C9 bonding regions at MCDO **1** and MVE **15**, respectively.

**Figure 7:**
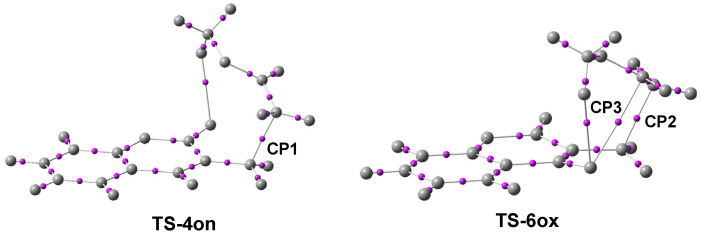
Positions of the CPs in **TS-4on** and **TS-6on,** with (3, +1) in gray and (3, −1) in pink.

Next, a QTAIM analysis of the electronic structure of **TS-4on** and **TS-6on** was performed. The positions of the (3, +1) and (3, −1) critical points (CPs) in both TSs are represented in Figure 7, while the calculated QTAIM parameters of the (3, −1) CPs characterizing the C1–C8 and C9–O regions are given in Table 3.

A comparative QTAIM analysis of the C1–C8 and C9–O interacting regions in **TS-4on** and **TS-6on** shows a high degree of electronic similarity between the two TSs (see Figure 7). The three selected CPs, **CP1**–**CP3**, show a positive Laplacian ∇^2^*ρ*(r) value, indicating the absence of any covalent interaction (see Table 3). **TS-4on** does not have any CP in the C9–O4 interacting region due to the large distance between these centers, while **TS-6on** shows the presence of **CP3** but with a very low electron density of 0.0078 e, without any chemical meaning.

Consequently, both the ELF and QTAIM topological analyses of the electron density of the diastereomeric **TS-4on** and **TS-6on** show a high degree of electronic similarity between them. This behavior is a consequence of the fact that the two TSs are only associated with the formation of the C1–C8 single bond. These findings account for the closer total electronic energy calculated between the two TSs, explaining the low *pseudocyclic* selectivity [31] of this HDA reaction under kinetic control.

### 2.5. RIAE Analysis of the HDA Reactions of MCDO ***1*** with MVE ***15*** and with Ethylene ***8***

To define the electronic effects responsible for the activation energies of the polar HDA reaction of MCDO **1** with MVE **15**, as well as the low *pseudocyclic* selectivity of this HDA reaction under kinetic control, an RIAE analysis [29] on the most favorable gas phase structures, **TS-4on** and **TS-6on**, was performed. As the reaction reference, the HDA reaction of MCDO **1** with ethylene **8** was also studied (see Section S1 in the Supplementary Materials). The B3LYP/6-311G(d,p) ξ$E_{\mathrm{total}}^{X}$ total, ξ$E_{\mathrm{intra}}^{X}$ intra-atomic, and ξ$E_{\mathrm{inter}}^{X}$ interatomic energies of the MCDO and the MVE and ethylene (Et) frameworks of the TSs with respect to the separated reagents are given in Table 4.

The RIAE analysis of **TS-4** and **TS-6** associated with the HDA reaction of MCDO **1** with ethylene **8** shows there is a close similarity between the two diastereomeric TSs. The ξ$E_{\mathrm{total}}^{X}$ energy analysis of the two interacting frameworks at the most favorable **TS-4** shows that while the Et framework is destabilized, ξ$E_{total}^{Et}$ = 41.06 kcal·mol^−1^, the electrophilic MCDO one is stabilized, ξ$E_{total}^{MCDO}$ = −23.87 kcal·mol^−1^. An analysis of the ξ$E_{intra}^{X}$ intra-atomic and ξ$E_{inter}^{X}$ interatomic energies at the two frameworks indicates that the intramolecular interaction at the electrophilic MCDO framework is the most stabilizing factor, with ξ$E_{intra}^{MCDO}$= −83.78 kcal·mol^−1^, while those at the Et framework are destabilizing, with ξ$E_{intra}^{Et}$= 44.92 kcal·mol^−1^. An analysis of the ξ$E_{intra}^{Et}$ intramolecular energies of the ethylene framework at **TS-6**, which are 2.33 kcal·mol^−1^ more energetic than those at **TS-4**, shows that these intramolecular energies are the main factor responsible for the *pseudocyclic* selectivity of this HDA reaction. It is noted that the difference between the two RIAE activation energies was found to be 1.83 kcal·mol^−1^.

An RIAE analysis of **TS-4on** and **TS-6on** associated with the HDA reaction of MCDO **1** with MVE **15** shows there is a great similarity between the two diastereomeric TSs (see Table 4), which is in complete agreement with the previous topological analysis of their electronic structure.

The analysis of the ξ$E_{total}^{X}$ energies of the two frameworks at the most favorable **TS-4on** shows that while the nucleophilic MVE framework is destabilized, ξ$E_{total}^{Et}$ = 41.56 kcal·mol^−1^, the electrophilic MCDO framework is stabilized, ξ$E_{total}^{MVE}$ = −37.60 kcal·mol^−1^. Interestingly, while the ξ$E_{total}^{Et}$ and ξ$E_{total}^{MVE}$ total energies in the HDA reactions with ethylene **8** and MVE **15** are very similar, the ξ$E_{total}^{MCDO}$ total energy in the reaction involving MVE **15** is ca. 14 kcal·mol^−1^ more negative than that in the reaction involving ethylene **8**. This behavior is a consequence of the higher GEDT happening at **TS-4on**, with 0.38 e, than that at the **TS-4**, with 0.23 e (see Section S1 in the Supplementary Materials).

The analysis of the ξ$E_{intra}^{X}$ intra-atomic and ξ$E_{inter}^{X}$ interatomic energies at the two frameworks indicates that the intramolecular interaction at the electrophilic MCDO framework is the more stabilizing factor, with ξ$E_{intra}^{MCDO}$= −66.20 kcal·mol^−1^, while the intramolecular interactions at the MVE framework are destabilizing, with ξ$E_{intra}^{MVE}$= 71.48 kcal·mol^−1^. Although the ξ$E_{intra}^{MCDO}$ intramolecular energies in the reaction involving MVE **15** are less stabilizing than those in the reaction involving ethylene **8**, the unfavorable ξ$E_{inter}^{MVE}$ inter-molecular energies in the reaction with MVE **15** are half those in the reaction with ethylene **8**. These behaviors are responsible for the strong reduction in the activation energies of the polar HDA reaction involving the nucleophilic MVE **15** with respect to that of ethylene **8**.

A similar result was obtained in the RIAE analysis of the stereoisomeric **TS-6on**. In fact, the very low difference between the RIAE activation energies associated with **TS-4on** and **TS-6on**, ΔΔE_act_ = 0.06 kcal·mol^−1^, accounts for the very low *pseudocyclic* selectivity in this HDA reaction under kinetic control.

**Figure 8:**
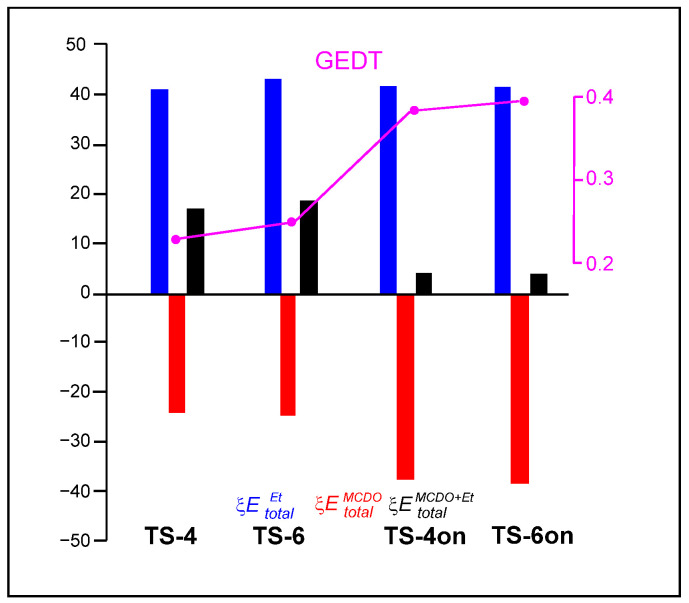
Graphical representation of the sets of the ξ$E_{total}^{Et}$, ξ$E_{total}^{MCDO}$, and ξ$E_{total}^{MCDO+Et}$ energies in that order for the TSs associated with the HDA reactions of MCDO **1** with ethylene **8** and MVE **15**. The ξ$E_{total}^{MCDO+Et}$ energies correspond to the RIAE activation energies of these HDA reactions. ξ$E_{total}^{X}$ energies of the Et and MCDO frameworks at the four TSs are shown in blue and red, respectively. The black bar represents the ξ$E_{total}^{MCDO+Et}$ energies. The GEDT values at the TSs are given in pink. ξ$E_{total}^{X}$ energies are given in kcal mol^−1^, and the GEDT is given in average number of electrons, e.

Finally, Figure 8 displays a graphical representation of the ξ$E_{total}^{X}$ energies of the MCDO framework and the ethylene and MVE frameworks at the two pairs of diasteromeric TSs, in blue and red, respectively. The ξ$E_{total}^{MCDO+Et}$ energies, shown in black, correspond to the RIAE activation energies of these DA reactions. As can be see, the stabilization of the MCDO framework with the increase in GEDT is the main factor responsible for the reduction in the RIAE activation energies. It is worth noting that at the four TSs, the ξ$E_{total}^{Et}$ is ca. 40 kcal·mol^−1^.

The ξ$E_{total}^{MCDO}$ total energies associated with the MCDO framework of **TS-6** and **TS-6on** are more stabilized than those at **TS-4** and **TS-4on** as a consequence of the slightly more polar character of the former, but the ξ$E_{total}^{Et}$ total energies associated with the Et and MVE frameworks of the former TSs are more destabilized. As a consequence, these HDA reactions are poorly *O4 pseudocyclic* selective under kinetic control (see Scheme 1).

## 3. Conclusions

The HDA reaction between MCDO **1** and the non-symmetric MVE **15**, experimentally studied by Appendino et al. [16], was investigated within the MEDT at the B3LYP/6-311(d,p) computational level. The DFT-based reactivity indices analysis of the two reagents indicates that this HDA reaction, classified as REDF, has a high polar character as a consequence of the strong electrophilic character of MCDO **1** and the strong nucleophilic character of MVE **15**. The analysis of the electrophilic Parr functions indicates that 55% of the electrophilic character of MCDO **1** is concentrated in the methylene C1 carbon, justifying the total *ortho* regioselectivity experimentally observed.

The analysis of the eight competitive reaction pathways indicates that this HDA reaction is totally *ortho* regioselective, *endo* selective, and poorly *pseudocyclic* selective. Due to the high polar character of this HDA reaction, it presents a very low activation energy of only 4.21 kcal·mol^−1^. The analysis of the relative Gibb free energies involved in the more favorable *ortho* reaction paths indicates that the complete *pseudocyclic* selectivity experimentally observed can only be explained by a thermodynamic control yielding the thermodynamically most stable CA **4** in Scheme 1.

The geometrical parameter analysis of the four *ortho* TSs shows the similitude between them, with a diastereomeric relationship. These TSs are characterized by a highly asynchronous C–C single bond formation process associated with a two-center interaction between the most nucleophilic C8 carbon of MVE **15** and the most electrophilic C1 carbon of MCDO **1**.

ELF and QTAIM topological analyses of the electronic structure of the diastereomeric **TS-4on** and **TS-6on** demonstrated the high degree of similarity in terms of electron density between them. Finally, an RIAE analysis of these TSs indicates that the stabilization of the electrophilic MCDO framework at these polar TSs, resulting from the high GEDT, is the main factor responsible for the acceleration found in this HDA reaction. A comparative RIAE analysis of the two diastereomeric **TS-4on** and **TS-6on** structures shows the great degree of similarity between them, accounting for their low levels of *pseudocyclic* selectivity under kinetic control.

## Figures and Tables

**Table 1:** B3LYP/6-31G* reactivity indices including electronic chemical potential μ, chemical hardness η, electrophilicity ω, and nucleophilicity *N*, in eV, for MCDO **1**, MVE **15,** and ethylene **8**.

Reactants	μ	η	ω	*N*
MCDO **1**	−4.88	4.16	2.87	2.16
ethylene **8**	−3.37	7.77	0.73	1.86
MVE **15**	−2.43	6.98	0.42	3.20

**Table 2:** B3LYP/6-311G(d,p) relative energies, ΔE in kcal·mol^−1^, in gas phase and in dioxane of the stationary points involved in the HDA reaction of MCDO **1** with MVE **15**.

	Gas Phase	Dioxane
**TS-4on**	4.21	3.48
**TS-4ox**	4.88	4.65
**TS-4mn**	19.76	20.05
**TS-4mx**	23.09	23.03
**TS-6on**	4.26	3.55
**TS-6ox**	5.44	5.15
**TS-6mn**	22.19	22.49
**TS-6mx**	25.69	25.60
**CA-4on**	−34.12	−33.62
**CA-4ox**	−35.39	−34.79
**CA-4mn**	−27.26	−26.61
**CA-4mx**	−26.64	−26.35
**CA-6on**	−19.02	−18.26
**CA-6ox**	−18.46	−18.07
**CA-6mn**	−26.31	−25.64
**CA-6mx**	−27.28	−26.62

**Table 3:** QTAIM parameters of the CPs (3, −1) associated with the C1–C8 and C9–O regions of **TS-4on** and **TS-6on**. CPs are given in a.u.

	TS-4on	TS-6on	
	CP1	CP2	CP3
Density *ρ*(r)	0.0850	0.0810	0.0078
Laplacian ∇^2^*ρ*(r)	0.0128	0.0194	0.0343
G(r)	0.0309	0.0300	0.0074
K(r)	0.0277	0.0251	0.0012
V(r)	−0.0587	−0.0552	−0.0062
E(r)	−0.0277	−0.0251	0.0012

**Table 4:** B3LYP/6-311G(d,p) gas-phase ξ$E_{\mathrm{total}}^{X}$ total energy, ξ$E_{\mathrm{intra}}^{X}$ intra-atomic energy, and ξ$E_{\mathrm{inter}}^{X}$ interatomic energy in kcal·mol^−1^ of the MCDO and the ethylene frameworks at the TSs with respect to those at the separated reagents. The sum of the ξ$E_{\mathrm{total}}^{X}$ energies of the two frameworks, i.e., ξ$E_{\mathrm{total}}^{\mathrm{MCDO+Et}}$, yields the RIAE activation energies.

	ƒ(X)	ξ$E_{\mathrm{intra}}^{X}$	ξ$E_{\mathrm{inter}}^{X}$	ξ$E_{\mathrm{total}}^{X}$	ξ$E_{\mathrm{intra}}^{\mathrm{MCDO+Et}}$
**TS-4**	MCDO	−83.78	59.90	−23.87	17.19
	ethylene	44.92	−3.87	41.06	
**TS-6**	MCDO	−84.44	59.89	−24.55	19.02
	ethylene	47.25	−3.68	43.57	
**TS-4on**	MCDO	−66.20	28.60	−37.60	3.96
	MVE	71.48	−29.92	41.56	
**TS-6on**	MCDO	−67.42	29.43	−37.99	4.02
	MVE	71.61	−29.59	42.02	

## Data Availability

The data that support the findings of this study are available from the corresponding author upon reasonable request.

## Supplementary Materials

The following supporting information can be downloaded at: https://www.mdpi.com/article/10.3390/molecules29215109/s1, Study of the HDA reaction between MCDO **1** and ethylene **8**. Scheme S1: Competitive reaction paths associated with the HDA reaction between MCDO **1** and ethylene **8**. B3LYP/6-311G(d,p) Relative energies in kcal·mol^−1^ are given in parenthesis; Figure S1: B3LYP/6-311G(d,p) geometries of TSs associated with HDA reaction between MCDO **1** and ethylene **2**; Figure S2: ELF Basin attractor positions and populations of the most relevant valence basins of **TS-4** and **TS-6**. Valence basin populations are given in average number of electrons, e. Theoretical background of the RIAE analysis. Computational Details. Figure S3: B3LYP/6-311G(d,p) Geometries of the meta regioisomeric TSs associated with the HDA reaction of MCDO **1** with MVE **15**. Distances are given in Angstroms Å. Table S1: B3LYP/6-311G(d,p)Total electronic energies, E in a.u., in gas phase and in dioxane, of the stationary points associated with the HDA reaction between MCDO **1** and MVE **15.** Table S2: B3LYP/6-311G(d,p) gas phase total, E in a.u., and relative, ΔE in kcal·mol^−1^, electronic energies of the stationary points associated with the more favorable pseudocyclic selective reaction paths of the HDA reaction between MCDO **1** and EVE **2**. Table S3: Thermodynamic data, H and G in kcal·mol^−1^ and S kcal·mol^−1^·K, computed at 101.1 °C in dioxane, of the stationary points associated with the ortho regioisomeric reaction paths of the HDA reaction between MCDO **1** and MVE **15**. Table S4. Total electronic energies, E in a.u., of the stationary points associated with the HDA reaction between MCDO **1** and ethylene **8**. Cartesian coordinates of the stationary points associated with the more favorable *pseudocyclic* selective reaction paths of the HDA reaction between MCDO **1** and EVE **2** [43,44,45,46,47,48,49,50,51,52,53,54,55,56,57,58,59,60,61,62].
