# Supplementary material for: An MEDT Study of the Reaction Mechanism and Selectivity of the Hetero-Diels–Alder Reaction Between 3-Methylene-2,4-Chromandione and Methyl Vinyl Ether
Source: Molecules. 2024 Oct 29;29(21):5109. doi: 10.3390/molecules29215109 (PMC11547322; doi:10.3390/molecules29215109)
Supplement: Supplementary file 1 [file molecules-29-05109-s001.zip › molecules-3238510-supplementary.pdf]

Supplementary Material

An MEDT Study of the Reaction Mechanism and Selectivity of the Hetero-Diels–Alder Reaction Between 3-Methylene-2,4-Chromandione and Methyl Vinyl Ether

Abderrazzak Bouhaoui [1], Aziz Moumad [1], Luis R. Domingo [2,*] and Latifa Bouissane [1,*]

[1] Molecular Chemistry, Materials and Catalysis Laboratory, Faculty of Sciences and Technologies, Sultan Moulay Slimane University, BP 523, Beni-Mellal 23000, Morocco; abderazakbohaoui@gmail.com (A.B.); azizmoumad2@gmail.com (A.M.)
[2] Department of Organic Chemistry, University of Valencia, Dr. Moliner 50, 46100 Burjassot, Valencia, Spain
* Correspondence: luisrdomingo@gmail.com (L.R.D.); l.bouissane@usms.ma (L.B.)

Index

S2 Study of the HDA reaction between of MCDO **1** and ethylene **8**.
S4 Theoretical background of the Relative Interacting Atomic Energy (RIAE) Analysis.
S5 Computational Details.
S6 **Figure S3** with the geometries of the *meta* regioisomeric TSs associated with the HDA reaction of MCDO **1** with MVE **15**.
S7 **Table S1** with the total electronic energies, in gas phase and in dioxane, of the stationary points associated with the HDA reaction between MCDO **1** and MVE **15**.
S7 **Table S2** with the gas phase total and relative electronic energies of the stationary points associated with the more favorable *pseudocyclic* selective reaction paths of the HDA reaction between MCDO **1** and EVE **2**.
S8 **Table S3** with the thermodynamic data of the stationary points associated with the *ortho* regioisomeric reaction paths of the HDA reaction between MCDO **1** and MVE **15**.
S9 **Table S4** with total electronic energies of the stationary points associated with the HDA reaction between MCDO **1** and ethylene **8**.
S10 B3LYP/6-311G(d,p) gas phase computed total energies and Cartesian coordinates of the of the stationary points associated with the HDA reaction between MCDO **1** and MVE **15**.
S20 B3LYP/6-311G(d,p) gas phase computed total energies and Cartesian coordinates of the of the stationary points associated with the HDA reaction between MCDO **1** and ethylene **8**.
S23 B3LYP/6-311G(d,p) gas phase computed total energies and Cartesian coordinates of the of the stationary points associated with the more favorable *pseudocyclic* selective reaction paths of the HDA reaction between MCDO **1** and EVE **2**.

Study of the HDA reaction between of MCDO 1 and ethylene 8

Due to the presence of two heterodiene frameworks in MCDO **1** two competitive reaction paths are feasible along the HDA reaction of MCDO **1** with ethylene **8**, namely *O4* and *O6* (see Scheme S1). Analysis of the stationary points located along the two competitive reaction paths indicates that this HDA reaction proceeds according to a non-concerted one-step mechanism. Relative B3LYP/6-311G(d,p) electronic energies in the gas phase are given in Scheme S1, while the total electronic energies are given in Table S3.

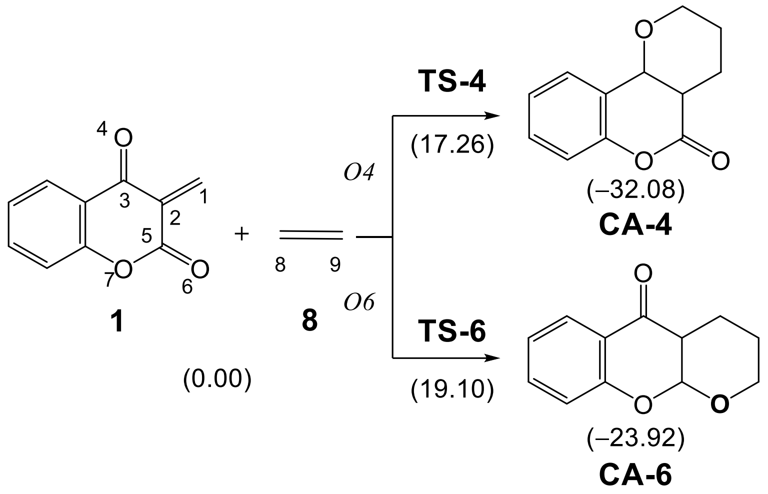

Scheme S1. Competitive reaction paths associated with the HDA reaction between MCDO **1** and ethylene **8**. B3LYP/6-311G(d,p) Relative energies in kcal·mol^{-1} are given in parenthesis.

The activation energy associated to this HDA reaction via **TS-4** is 17.26 kcal·mol^{-1}; formation of **CA-4** is exothermic by 32.08 kcal·mol^{-1}. This reaction is *pseudocyclic O4* selective [31] as **TS-6** is located 1.83 kcal·mol^{-1} above **TS-4**. The high activation energy associated to this HDA reaction is a consequence of the marginal nucleophilic character of ethylene **8**; *N* = 1.86 eV (see Table 1).

The geometries of two TSs associated with HDA reaction between MCDO **1** and ethylene **8** are given in Figure S1. The distances between the two pairs of interacting atoms are 2.010 Å (C1–C8) and 2.304 Å (O4–C9) at **TS-4**, and 1.942 Å (C1–C8) and 2.320 Å (O6–C9) at **TS-6**. The more favorable **TS-4** is slightly more delayed and less asynchronous (see later). In the two TSs, the shorter C1–C8 distances involve the most electrophilic C1 carbon of MCDO **1**.

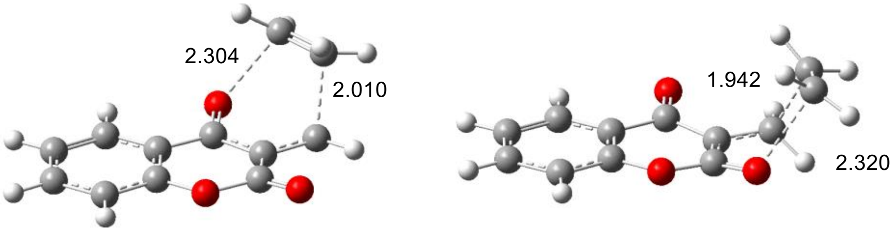

TS-4 **TS-6**

Figure S1. B3LYP/6-311G(d,p) geometries of TSs associated with HDA reaction between MCDO **1** and ethylene **2**.

Figure S2 shows the ELF basin attractor positions, together with the most relevant valence basin populations at **TS-4** and **TS-6**. While **TS-4** shows the presence of two V(C1) and V(C8) monosynaptic basins, integrating 0.13 and 0.40e, respectively, **TS-6** shows the presence of a V(C1,C8) disynaptic basin integrating 0.70e. Thus, while the ELF of **TS-4** shows the presence of the two C1 and C8 *pseudoradical* centers required for the subsequent formation of the first C1–C8 single bond [17], the ELF of **TS-6** indicates that the formation of the first C1–C8 single bond has begun, a C–C distance of 1.92 Å.

Neither monosynaptic nor disynaptic basin are observed in the O4 – C9 or O6 – C9 regions, indicating that the two TSs are only associated with the C1–C8 single bond formation. ELF topology of **TS-4** and **TS-6** indicates the highly asynchronous character of these stereoisomeric TS.

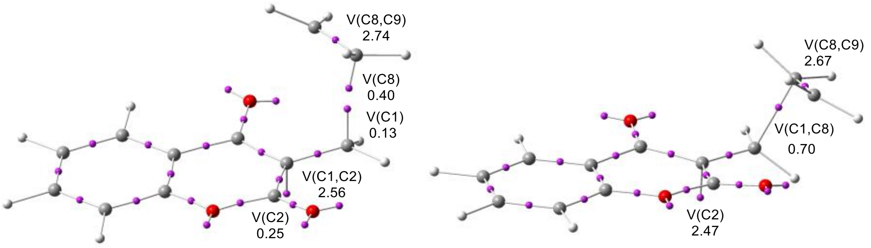

TS-4 **TS-6**

Figure S2. ELF Basin attractor positions and populations of the most relevant valence basins of **TS-4** and **TS-6**. Valence basin populations are given in average number of electrons, e.

Finally, the global electron density transfer [17] (GEDT) was computed at the two TSs. The values of the GEDT at the two TSs, 0.23 e at **TS-4** and 0.25 at **TS6**, indicate that this HDA reaction has a high polar character, the reaction being classified of reverse electron density flux [38] (REDF). These high GEDT values is a consequence of the strong electrophilic character of MCDO **1**.

Theoretical background of the RIAE Analysis

The Interacting Quantum Atoms [28] IQA, based on the Quantum Theory of Atoms in Molecules [25,26] (QTAIM), divides the E_{total}^{IQA} total energy into two main energy contributions: the E_{intra}^{A} intra-atomic energies and the E_{inter}^{AB} interatomic energies (see Equation S1). The E_{inter}^{AB} energies are, in turn, divided into four additional electrostatic terms: the V_{ne}^{AB} and V_{en}^{AB} nuclei-electron interactions, the V_{ee}^{AB} electron-electron interactions, and the V_{nn}^{AB} nuclei-nuclei interactions (see Equation S3).

$$E_{total}^{IQA} = \sum E_{intra}^{A} + \sum E_{inter}^{AB} \tag{S1}$$

$$E_{intra}^{A} = T(A) + V_{ne}^{A} + V_{ee}^{A} \tag{S2}$$

$$E_{inter}^{AB} = ½V_{ne}^{AB} + ½V_{en}^{AB} + ½V_{ee}^{AB} + V_{nn}^{AB} \tag{S3}$$

Thanks to the additivity of the topological atoms [43], an IQF approach has been recently introduced [44], which allows the grouping of the IQA energy in terms of convenient fragments of the system, allowing a more chemically meaningful analysis of the interactions that take place between the atoms forming groups. In this sense, in the RIAE analysis of P-DA reactions, the atoms belonging to the TSs are regrouped in the two interacting frameworks f(X) related to the diene and ethylene reagents [30,45].

By default, the sum of all IQA atomic energies belonging to the considered framework f(X) (where X represents the diene or ethylene frameworks) at the TSs, and those of the separated reagents at the ground states (GSs), are computed. The RIAEs, i.e., the relative ξE_{total}^{X} total, ξE_{intra}^{X} intra-atomic, and ξE_{inter}^{X}interatomic energies, are obtained using Equations S3-S5. The symbol ξ denotes the IQA energy differences between the GS and the TS states of the two-interacting frameworks f(X); i.e. f(diene) and f(ethylene) in DA reactions.

$$\xi E_{total}^{X} = \xi E_{intra}^{X} + \xi E_{inter}^{X} \tag{S4}$$

$$\xi E_{intra}^{X} = \sum E_{intra}^{X(TS)} - \sum E_{intra}^{X(GS)} \tag{S5}$$

$$\xi E_{inter}^{X} = \sum E_{inter}^{X(TS)} - \sum E_{inter}^{X(GS)} \tag{S6}$$

The herein proposed RIAE analysis provides a measure of how much the two interacting frameworks f(X) are destabilized (resulting in positive relative energies) or stabilized (resulting in negative relative energies) when going from their GS to the TS. The sum of the ξE_{total}^{X} energies of the two interacting frameworks, $\xi E_{total}^{diene+ethylene}$, provides the RIAE activation energy of DA reactions obtained through the present EDA [30,45].

Computational Details

The DFT B3LYP functional [46,47] together with the standard 6-311G(d,p) basis set [48], which includes d-type polarization for second-row elements and p-type polarization functions for hydrogens, was used throughout this Molecular Electron Density Theory (MEDT) [22] study. The TSs were characterized by the presence of only one imaginary frequency. The Berny method was used in optimizations [49,50]. The intrinsic reaction coordinates [51] (IRC) paths were traced to obtain the energy profiles connecting each TS to the two associated minima in the potential energy surface [52,53].

Solvent effects of 1,4-dioxane were taken into account by full optimization of the gas phase structures at the same computational level using the polarizable continuum model [54,55] (PCM) in the framework of the self-consistent reaction field [56-58] (SCRF). Values of B3LYP/6-311G(d,p) enthalpies, entropies, and Gibbs free energies in dioxane were calculated with standard statistical thermodynamics [48] at 101.1 ºC and 1 atm by PCM frequency calculations at the solvent-optimized structures.

The GEDT [17] values were computed using the equation GEDT(f) = Σq_f, where q are the natural charges [33,34] of the atoms belonging to one of the two frameworks (f) at the TS geometries. The global and local DFT-based reactivity indices [19,20] were calculated by using the equations given in reference [20], using the B3LYP/6-31G(d) computational level because the original nucleophilicity and electrophilicity scales were established at that level [20].

The Gaussian 16 suite of programs was used to perform the calculations [59]. Electron localization function [23] (ELF) analyses of the B3LYP/6-311G(d,p) monodeterminantal wave functions were implemented using the TopMod [60] package with a cubical grid of step size of 0.1 Bohr. The QTAIM [25,26] and IQA [28] analysis were performed with the AIMAll package [61] using the corresponding gas phase B3LYP/6-311G(d,p) monodeterminantal wave functions. Molecular geometries, ELF basin attractors and QTAIM critical points were visualized using the GaussView program [62].

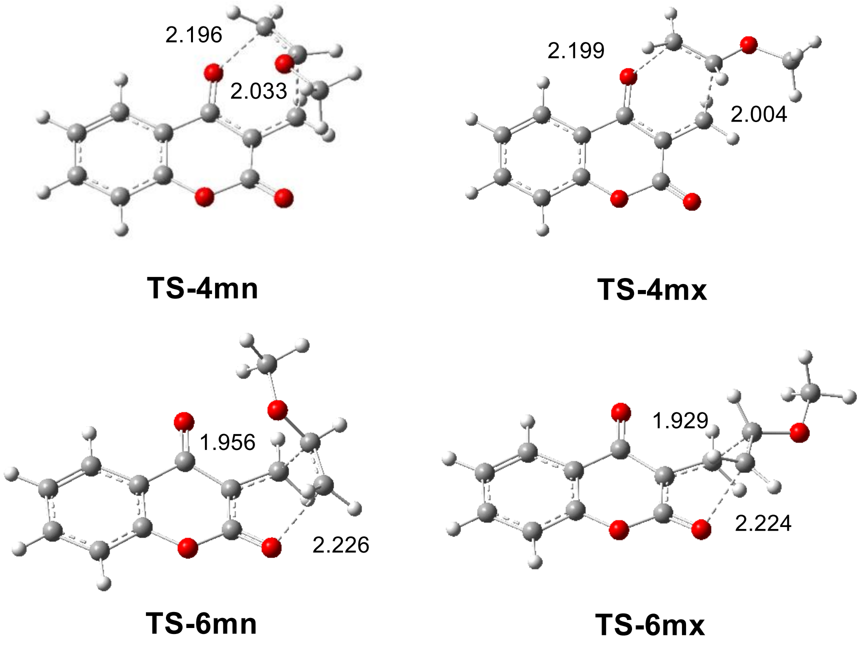

Figure S3. B3LYP/6-311G(d,p) Geometries of the *meta* regioisomeric TSs associated with the HDA reaction of MCDO **1** with MVE **15**. Distances are given in Angstroms Å.

Table S1. B3LYP/6-311G(d,p)Total electronic energies, E in a.u., in gas phase and in dioxane, of the stationary points associated with the HDA reaction between MCDO **1** and MVE **15**.

	Gas phase	*Dioxane*
MCDO **1**	-610.495019	-610.499124
MVE **15**	-193.168477	-193.170223
**TS-4on**	-803.656793	-803.663802
**TS-4ox**	-803.655723	-803.661934
**TS-4mn**	-803.632010	-803.637391
**TS-4mx**	-803.626706	-803.632647
**TS-6on**	-803.628129	-803.633506
**TS-6ox**	-803.622564	-803.628549
**TS-6mn**	-803.656715	-803.663684
**TS-6mx**	-803.654832	-803.661146
**CA-4on**	-803.717877	-803.722924
**CA-4ox**	-803.719892	-803.724792
**CA-4mn**	-803.706940	-803.711760
**CA-4mx**	-803.705953	-803.711331
**CA-6on**	-803.693803	-803.698447
**CA-6ox**	-803.692922	-803.698146
**CA-6mn**	-803.705426	-803.710214
**CA-6mx**	-803.706976	-803.711762

Table S2. B3LYP/6-311G(d,p) gas phase total, E in a.u., and relative, ΔE in kcal·mol^{-1}, electronic energies of the stationary points associated with the more favorable *pseudocyclic* selective reaction paths of the HDA reaction between MCDO **1** and EVE **2**.

	E	ΔE
MCDO **1**	-610.495019	
EVE **2**	-232.498721	
**TS-Et-4mn**	-842.987349	4.01
**TS-Et-6on**	-842.987389	3.99
**CA-Et-4mn**	-843.035528	-26.22
**CA-Et-6on**	-843.047989	-34.04

Table S3. Thermodynamic data, H and G in kcal·mol^{-1} and S kcal·mol^{-1}·K, computed at 101.1 °C in dioxane, of the stationary points associated with the *ortho* regioisomeric reaction paths of the HDA reaction between MCDO **1** and MVE **15**.

	H	S	G
MCDO **1**	-610.346967	107.56	-610.411107
MVE **15**	-193.078917	71.23	-193.121393
**TS-4on**	-803.416941	137.86	-803.499153
**TS-4ox**	-803.415208	138.49	-803.497792
**TS-6mn**	-803.416786	137.86	-803.498994
**TS-6mx**	-803.414374	139.18	-803.497372
**CA-4on**	-803.472372	131.29	-803.550661
**CA-4ox**	-803.474074	130.51	-803.551903
**CA-6mn**	-803.459519	131.01	-803.537643
**CA-6mx**	-803.460847	130.27	-803.538527

Table S4. Total electronic energies, E in a.u., of the stationary points associated with the HDA reaction between MCDO **1** and ethylene **8**.

**8**	-78.613979
**1**	-610.495019
**TS-4**	-689.081490
**TS-6**	-689.078566
**CA-4**	-689.160119
**CA-6**	-689.147111

B3LYP/6-311G(d,p) gas phase computed total energies and Cartesian coordinates of the of the stationary points associated with the HDA reaction between MCDO **1** and MVE **15**.

MCDO **1**
E(RB3LYP) = -610.495019390 A.U.

6	0.624506	1.285816	-0.000095
6	1.828826	0.392567	0.000074
6	1.691098	-1.093671	-0.000188
6	-0.715190	-0.813850	-0.000165
6	-0.671866	0.584325	-0.000084
8	0.725718	2.498912	-0.000029
8	2.604989	-1.866903	0.000104
8	0.411445	-1.605728	-0.000346
6	3.057395	0.923392	0.000464
1	3.934285	0.288532	0.000632
1	3.171766	2.000695	0.000658
6	-1.877748	1.301120	0.000056
6	-1.932659	-1.491274	-0.000105
6	-3.114290	-0.762173	0.000034
6	-3.092536	0.637762	0.000114
1	-1.817890	2.382662	0.000117
1	-1.928262	-2.573738	-0.000173
1	-4.061783	-1.288654	0.000085
1	-4.020550	1.196169	0.000226

MVE **15**
E(RB3LYP) = -193.168477167 A.U.

6	-1.876125	-0.070988	0.000017
6	-0.615411	0.351413	0.000011
1	-2.114374	-1.127002	0.000002
1	-2.685234	0.645540	0.000037
1	-0.362323	1.410487	0.000026
8	0.443343	-0.502866	-0.000016
6	1.726250	0.109698	-0.000012
1	2.455561	-0.698943	-0.000036
1	1.875667	0.726078	-0.894376
1	1.875681	0.726036	0.894378

TS-4mn
E(RB3LYP) = -803.632009870 A.U.

6	3.717317	-1.173176	-0.510882
6	4.050137	0.176691	-0.677653
6	3.118712	1.173070	-0.419343
6	1.841538	0.815881	0.011733
6	1.494147	-0.528154	0.193273
6	2.447730	-1.519024	-0.077184
8	0.954064	1.839020	0.221911
6	-0.355488	1.620623	0.628572
6	-0.711022	0.251550	0.981827
6	0.150388	-0.840336	0.689395
8	-0.277713	-2.022933	0.781924
6	-2.030209	-0.054622	1.357345
8	-1.088063	2.579598	0.674548
1	4.451670	-1.942648	-0.717021
1	5.043623	0.451815	-1.013008

1	3.351946	2.222437	-0.548795
1	2.160253	-2.552960	0.070119
1	-2.176323	-0.899570	2.016782
1	-2.705926	0.780426	1.503264
6	-2.220438	-2.204487	-0.224599
6	-2.838111	-0.961438	-0.273306
8	-2.586595	-0.207704	-1.387709
1	-1.604447	-2.542969	-1.043636
1	-2.524460	-2.929705	0.514051
1	-3.832515	-0.860761	0.162368
6	-3.293183	1.031548	-1.468398
1	-3.124994	1.411523	-2.474725
1	-4.368765	0.875894	-1.318783
1	-2.912714	1.753912	-0.740691

TS-4mx

E(RB3LYP) = -803.626706429 A.U.

6	3.863509	-1.552346	0.058838
6	4.408603	-0.289071	0.319165
6	3.603547	0.841901	0.330252
6	2.238715	0.709783	0.075393
6	1.678253	-0.545147	-0.195508
6	2.507309	-1.675314	-0.196617
8	1.488991	1.855500	0.127116
6	0.112842	1.863319	-0.086302
6	-0.484214	0.600206	-0.506004
6	0.243684	-0.617499	-0.488978
8	-0.368930	-1.713410	-0.621400
6	-1.870841	0.511843	-0.729078
8	-0.467226	2.907311	0.072474
1	4.501192	-2.428215	0.053777
1	5.469828	-0.187735	0.515950
1	4.003197	1.826933	0.536005
1	2.054158	-2.636186	-0.408452
1	-2.203237	-0.198161	-1.475491
1	-2.425448	1.441217	-0.667516
6	-2.036570	-1.684066	0.811574
6	-2.860060	-0.578207	0.631119
8	-4.071874	-0.813976	0.013402
1	-1.241948	-1.656195	1.538847
1	-2.333358	-2.647698	0.424272
1	-2.862420	0.198730	1.393482
6	-5.027622	0.234704	0.127417
1	-5.934923	-0.124181	-0.355725
1	-5.244641	0.458476	1.178740
1	-4.693018	1.148988	-0.374250

TS-4on

E(RB3LYP) = -803.656793029 A.U.

6	3.464538	-1.400636	0.295867
6	3.946948	-0.293381	-0.412498
6	3.122552	0.791147	-0.678567
6	1.800425	0.772611	-0.231116
6	1.300065	-0.328063	0.475226
6	2.149530	-1.412312	0.732957
8	1.037059	1.866168	-0.535760
6	-0.292236	1.982028	-0.126750

6	-0.819503	0.915542	0.706684
6	-0.100902	-0.311826	0.940275
8	-0.637010	-1.301863	1.469791
6	-2.130186	1.031368	1.196086
8	-0.896398	2.958502	-0.499580
1	4.117947	-2.239643	0.503894
1	4.974901	-0.276631	-0.756883
1	3.475255	1.657002	-1.224858
1	1.742985	-2.246534	1.292427
1	-2.572912	2.020225	1.176879
1	-2.379699	0.405696	2.042939
6	-3.474967	0.280260	-0.073498
6	-3.014084	-0.999114	-0.302702
8	-2.124447	-1.208542	-1.245734
1	-3.339530	1.012142	-0.861238
1	-4.366988	0.377406	0.532737
1	-3.275996	-1.838872	0.335752
6	-1.508782	-2.516305	-1.290680
1	-0.823463	-2.491396	-2.133547
1	-0.972763	-2.682249	-0.354290
1	-2.273755	-3.279179	-1.451241

TS-4ox
E(RB3LYP) = -803.655723181 A.U.

6	-2.952397	1.183386	0.761551
6	-3.062247	-0.188141	0.817857
8	-3.952221	-0.819472	0.078338
1	-2.385644	1.680660	1.536239
1	-3.781437	1.734232	0.332450
1	-2.391325	-0.799711	1.412583
6	3.188928	-2.249878	0.072634
6	4.076280	-1.196831	0.327367
6	3.631505	0.117763	0.337360
6	2.285002	0.386634	0.086374
6	1.384676	-0.653786	-0.173674
6	1.853839	-1.974250	-0.173323
8	1.905960	1.700404	0.128853
6	0.591312	2.107302	-0.108338
6	-0.351685	1.072044	-0.505194
6	-0.026840	-0.329200	-0.445487
8	-0.899857	-1.207928	-0.576891
6	-1.665912	1.442156	-0.817001
8	0.351193	3.281589	0.024532
1	3.546731	-3.272601	0.066483
1	5.122729	-1.405198	0.519830
1	4.299722	0.946212	0.536343
1	1.138256	-2.761771	-0.376716
1	-1.862906	2.502826	-0.917617
1	-2.233083	0.776881	-1.455204
6	-3.828210	-2.258148	0.011195
1	-4.052250	-2.694482	0.987926
1	-4.567119	-2.584837	-0.716171
1	-2.819770	-2.513270	-0.312738

TS-6m
E(RB3LYP) = -803.656715136 A.U.

6	-3.964501	0.368206	-0.742823

6	-3.946142	-0.963890	-0.312101
6	-2.798254	-1.511665	0.244000
6	-1.657179	-0.719029	0.372537
6	-1.657908	0.615120	-0.047704
6	-2.825529	1.146026	-0.610581
8	-0.549238	-1.326745	0.910209
6	0.627604	-0.623579	1.126048
6	0.652204	0.778485	0.811621
6	-0.437280	1.439877	0.109379
8	-0.361257	2.591808	-0.317462
6	1.842665	1.477491	1.079827
8	1.582242	-1.267954	1.532354
1	-4.865425	0.786785	-1.175560
1	-4.834251	-1.577949	-0.411142
1	-2.761441	-2.540008	0.581587
1	-2.797857	2.180677	-0.931016
1	2.476947	1.089887	1.865835
1	1.773127	2.557467	1.007469
6	3.129929	1.332175	-0.408990
6	3.275545	-0.028611	-0.594668
8	2.428269	-0.671515	-1.363856
1	2.546578	1.876889	-1.142499
1	3.975599	1.855329	0.020603
1	4.001750	-0.620925	-0.043387
6	2.471046	-2.118073	-1.334409
1	2.176379	-2.455767	-0.339083
1	1.759929	-2.448961	-2.086464
1	3.475339	-2.463367	-1.589120

TS-6mx
E(RB3LYP) = -803.654832342 A.U.

6	-4.431073	0.173710	0.396126
6	-4.257336	-1.214580	0.341201
6	-3.002207	-1.761680	0.113218
6	-1.910155	-0.911761	-0.063333
6	-2.063994	0.477606	-0.015877
6	-3.339177	1.007885	0.219367
8	-0.692394	-1.516773	-0.260360
6	0.448894	-0.769666	-0.487023
6	0.335338	0.658884	-0.571962
6	-0.892504	1.362500	-0.222275
8	-0.968009	2.585510	-0.120952
6	1.503652	1.379029	-0.866926
8	1.504173	-1.384750	-0.553214
1	-5.414908	0.591249	0.574564
1	-5.107599	-1.873224	0.478203
1	-2.844038	-2.832129	0.070933
1	-3.430821	2.086945	0.253041
1	2.250418	0.909092	-1.494165
1	1.366534	2.447420	-0.994315
6	2.749746	1.548610	0.713178
6	3.244383	0.268578	0.853113
8	4.297835	-0.122337	0.167092
1	2.050909	1.903136	1.458342
1	3.410599	2.289388	0.277289
1	2.751250	-0.478591	1.467284
6	4.586223	-1.540043	0.179253
1	5.408834	-1.679591	-0.517424
1	4.894058	-1.844879	1.182482

1	3.701191	-2.086463	-0.145117

TS-6on
E(RB3LYP) = -803.628128843 A.U.

6	3.911922	-0.996168	-0.275002
6	4.101799	0.343142	-0.640962
6	3.057793	1.252746	-0.562029
6	1.809523	0.818347	-0.114628
6	1.599852	-0.513996	0.260190
6	2.670057	-1.415662	0.169615
8	0.810286	1.759028	-0.086509
6	-0.422379	1.440160	0.430852
6	-0.680513	0.138587	0.928259
6	0.271674	-0.949810	0.744099
8	0.011048	-2.127747	0.989033
6	-2.010903	-0.102662	1.341697
8	-1.302617	2.307722	0.347087
1	4.734966	-1.697865	-0.339598
1	5.072161	0.676569	-0.990990
1	3.180355	2.291011	-0.843859
1	2.483444	-2.441784	0.462728
1	-2.507366	0.669724	1.915482
1	-2.212726	-1.116497	1.672380
6	-3.151829	1.408591	-0.505733
6	-3.118748	0.036544	-0.263812
8	-2.588041	-0.724183	-1.276023
1	-2.763461	1.816029	-1.426955
1	-3.729028	2.058519	0.133602
1	-3.979009	-0.394779	0.253224
6	-2.656545	-2.139236	-1.086050
1	-2.375450	-2.585614	-2.038676
1	-1.960142	-2.469776	-0.310270
1	-3.679391	-2.447593	-0.834750

TS-6ox
E(RB3LYP) = -803.622564130 A.U.

6	-4.266698	1.038047	0.329532
6	-4.489412	-0.344586	0.285680
6	-3.435420	-1.224793	0.089962
6	-2.144378	-0.717522	-0.061128
6	-1.900905	0.660843	-0.025822
6	-2.982151	1.530503	0.175099
8	-1.140863	-1.642829	-0.219575
6	0.146127	-1.224463	-0.465192
6	0.451962	0.153319	-0.550051
6	-0.525943	1.181193	-0.209537
8	-0.247141	2.372706	-0.104059
6	1.818611	0.460211	-0.753273
8	1.025097	-2.099504	-0.488568
1	-5.097552	1.716631	0.481709
1	-5.493333	-0.735676	0.406021
1	-3.583606	-2.296912	0.056586
1	-2.769061	2.592483	0.199631
1	2.357571	-0.137711	-1.478499
1	2.038653	1.523056	-0.768332
6	2.579391	-1.432136	0.955545
6	3.002707	-0.138224	0.646634

8	4.263157	-0.048149	0.076840
1	1.783056	-1.593942	1.663367
1	3.180869	-2.282493	0.668318
1	2.757541	0.651490	1.356866
6	4.843312	1.251291	0.088771
1	4.915275	1.642677	1.110847
1	5.845782	1.142012	-0.322110
1	4.279601	1.958495	-0.528844

CA-4mn
E(RB3LYP) = -803.706939859 A.U.

6	-3.689854	-1.706307	-0.154344
6	-4.356878	-0.475435	-0.188072
6	-3.647179	0.715009	-0.127471
6	-2.256138	0.675649	-0.035049
6	-1.572163	-0.548091	-0.000651
6	-2.308460	-1.742081	-0.059567
8	-1.599138	1.866347	0.024140
6	-0.207243	1.949826	0.106386
6	0.529480	0.698173	0.142777
6	-0.128837	-0.493515	0.100129
8	0.488746	-1.691998	0.133959
6	2.033785	0.779508	0.190340
8	0.282063	3.049923	0.135882
1	-4.253752	-2.630097	-0.200675
1	-5.437949	-0.447729	-0.260988
1	-4.139819	1.678764	-0.149959
1	-1.774885	-2.682898	-0.028906
1	2.364751	1.591531	-0.460744
1	2.379211	1.031004	1.200248
6	1.888941	-1.697330	0.468920
6	2.645089	-0.563961	-0.216485
8	3.993146	-0.718545	0.189271
1	1.996102	-1.606991	1.554669
1	2.265654	-2.667956	0.152486
1	2.562429	-0.693930	-1.306593
6	4.927969	0.030301	-0.571862
1	4.794545	1.111009	-0.444447
1	5.918306	-0.246185	-0.210662
1	4.855805	-0.212810	-1.640763

CA-4mx
E(RB3LYP) = -803.705952969 A.U.

6	3.476004	-1.705284	-0.301161
6	4.127635	-0.481294	-0.496790
6	3.429604	0.713790	-0.402071
6	2.065844	0.686077	-0.110974
6	1.397886	-0.530593	0.088688
6	2.122222	-1.729172	-0.009106
8	1.420070	1.881647	-0.027292
6	0.052553	1.974164	0.242318
6	-0.664085	0.731874	0.458645
6	-0.016442	-0.464375	0.392336
8	-0.614528	-1.653597	0.587753
6	-2.145917	0.815595	0.708348
8	-0.433252	3.076862	0.274306
1	4.030212	-2.632976	-0.377566

1	5.187200	-0.462609	-0.724481
1	3.910492	1.672605	-0.548729
1	1.600681	-2.664324	0.146651
1	-2.574595	1.601556	0.083723
1	-2.349163	1.119723	1.742183
6	-1.977107	-1.647996	1.057619
6	-2.809530	-0.539609	0.431194
8	-2.900639	-0.845260	-0.952338
1	-1.966290	-1.541730	2.147437
1	-2.372398	-2.625627	0.787971
1	-3.811254	-0.567451	0.886656
6	-3.888310	-0.117166	-1.660492
1	-3.910400	-0.523837	-2.671417
1	-4.880474	-0.242166	-1.203582
1	-3.659366	0.953551	-1.719594

CA-4on
E(RB3LYP) = -803.717876846 A.U.

6	2.495647	-2.609831	-0.120118
6	3.632474	-1.794778	-0.055587
6	3.508784	-0.415220	0.020335
6	2.236694	0.156831	0.029869
6	1.087087	-0.644350	-0.032783
6	1.233819	-2.038611	-0.106820
8	2.162550	1.513358	0.106858
6	0.943254	2.196983	0.105479
6	-0.264445	1.394523	0.033375
6	-0.192693	0.034607	-0.014770
8	-1.274326	-0.770148	-0.057138
6	-1.583295	2.118941	-0.017850
8	0.982014	3.399402	0.161914
1	2.602779	-3.686193	-0.179948
1	4.619996	-2.241451	-0.064765
1	4.371802	0.236425	0.071679
1	0.344152	-2.652284	-0.156826
1	-1.491201	2.986302	-0.675143
1	-1.829341	2.523519	0.971252
6	-2.686737	1.178674	-0.511095
6	-2.582847	-0.162945	0.185706
8	-3.539963	-1.022276	-0.303522
1	-2.598002	0.999466	-1.586144
1	-3.679763	1.591037	-0.322781
1	-2.660706	-0.059865	1.279494
6	-3.681110	-2.244731	0.419046
1	-4.544378	-2.754704	-0.005610
1	-2.794974	-2.875251	0.311515
1	-3.862372	-2.050241	1.483657

CA-4ox
E(RB3LYP) = -803.719891900 A.U.

6	-2.794326	0.624968	1.071528
6	-2.520105	-0.779787	0.562023
8	-2.988715	-0.874976	-0.742477
1	-2.496421	0.671145	2.123017
1	-3.868541	0.810126	1.016216
1	-2.960604	-1.556508	1.196894
6	2.930639	-2.173077	0.282294

6	3.860212	-1.181779	-0.055321
6	3.451249	0.126874	-0.266919
6	2.100284	0.449183	-0.138966
6	1.154909	-0.531404	0.195787
6	1.589194	-1.850078	0.405921
8	1.743323	1.744492	-0.354108
6	0.421009	2.185066	-0.244800
6	-0.579503	1.192280	0.107748
6	-0.224909	-0.106426	0.304001
8	-1.102095	-1.089674	0.623106
6	-2.006734	1.645801	0.245625
8	0.207952	3.352647	-0.447129
1	3.260084	-3.191942	0.446817
1	4.909713	-1.434437	-0.152948
1	4.150042	0.911349	-0.528453
1	0.856457	-2.601948	0.667970
1	-2.031758	2.632750	0.711695
1	-2.458995	1.757243	-0.745099
6	-2.947946	-2.185850	-1.301810
1	-3.499839	-2.896302	-0.672993
1	-3.428282	-2.120183	-2.276791
1	-1.919428	-2.535819	-1.424126

CA-6mn
E(RB3LYP) = -803.705426245 A.U.

6	-4.398178	-0.332337	0.033310
6	-3.965842	-1.661805	-0.068461
6	-2.612762	-1.962788	-0.112015
6	-1.692767	-0.917258	-0.051191
6	-2.096789	0.415007	0.049174
6	-3.469842	0.693378	0.090824
8	-0.360741	-1.264523	-0.090881
6	0.564512	-0.287360	-0.044065
6	0.294027	1.042075	0.036106
6	-1.084404	1.498758	0.098441
8	-1.392985	2.684482	0.173655
6	1.420606	2.042312	0.027043
8	1.777427	-0.838885	-0.095947
1	-5.458246	-0.110307	0.065921
1	-4.691579	-2.465596	-0.114265
1	-2.253096	-2.981079	-0.190631
1	-3.765624	1.732756	0.167149
1	1.552308	2.467892	1.028817
1	1.140457	2.881248	-0.613857
6	2.719815	1.394905	-0.462027
6	2.917278	0.044012	0.196100
8	4.044306	-0.565338	-0.296525
1	2.694975	1.232027	-1.543053
1	3.589106	2.016500	-0.238691
1	2.947292	0.121609	1.293830
6	4.421122	-1.768987	0.374704
1	3.678907	-2.555304	0.218785
1	5.374310	-2.073298	-0.054302
1	4.546489	-1.591804	1.450216

CA-6mx
E(RB3LYP) = -803.706975974 A.U.

6	-4.290590	-0.067886	-0.322976
6	-4.001526	-1.398359	0.010753
6	-2.698916	-1.792156	0.277352
6	-1.683883	-0.839058	0.205754
6	-1.944885	0.492113	-0.123287
6	-3.269796	0.865231	-0.388204
8	-0.406769	-1.276411	0.474863
6	0.604547	-0.389073	0.421011
6	0.475968	0.928588	0.124316
6	-0.835988	1.476489	-0.181968
8	-1.019850	2.657961	-0.458275
6	1.692428	1.814965	0.113687
8	1.743391	-1.026936	0.725964
1	-5.312675	0.227247	-0.528842
1	-4.799861	-2.129729	0.062469
1	-2.449369	-2.813204	0.537540
1	-3.454278	1.902288	-0.641424
1	2.016407	1.990274	-0.916991
1	1.424536	2.790315	0.525190
6	2.823365	1.168118	0.917942
6	2.986597	-0.289260	0.519037
8	3.371825	-0.351050	-0.810878
1	2.601090	1.204018	1.988427
1	3.775046	1.677056	0.753802
1	3.686444	-0.833187	1.162076
6	3.729088	-1.653766	-1.271338
1	4.065950	-1.534993	-2.299784
1	4.545331	-2.070008	-0.667428
1	2.874159	-2.334253	-1.240898

CA-6on
E(RB3LYP) = -803.693802632 A.U.

6	4.373650	0.812224	-0.091432
6	4.463734	-0.582780	-0.194811
6	3.320089	-1.367116	-0.182406
6	2.081046	-0.739855	-0.063605
6	1.963327	0.646965	0.040038
6	3.133902	1.417825	0.024088
8	0.973939	-1.558977	-0.050970
6	-0.242988	-0.998826	0.060226
6	-0.486496	0.334667	0.156197
6	0.623784	1.274361	0.152811
8	0.469601	2.489795	0.223916
6	-1.901796	0.849781	0.223169
8	-1.162411	-1.966379	0.043825
1	5.274859	1.413682	-0.102544
1	5.433744	-1.057824	-0.285507
1	3.362408	-2.446226	-0.260536
1	3.024561	2.492645	0.103840
1	-1.969992	1.751449	-0.389665
1	-2.160578	1.148838	1.245763
6	-2.504190	-1.568512	0.395703
6	-2.884201	-0.231438	-0.235088
8	-4.220153	-0.000932	0.174648
1	-2.579235	-1.498628	1.485046
1	-3.148263	-2.370239	0.041673
1	-2.838327	-0.333977	-1.330421
6	-4.883283	1.034175	-0.534214
1	-5.913980	1.045067	-0.180261

1	-4.434467	2.016661	-0.346972
1	-4.878010	0.841680	-1.615816

CA-6ox

E(RB3LYP) = -803.692921942 A.U.

6	-4.213392	0.659210	-0.326477
6	-4.211253	-0.724827	-0.548462
6	-3.036799	-1.454885	-0.444084
6	-1.860063	-0.784822	-0.113155
6	-1.834766	0.592558	0.112158
6	-3.034630	1.308197	-0.000086
8	-0.720538	-1.551203	-0.017083
6	0.438740	-0.948488	0.300662
6	0.589311	0.381249	0.535208
6	-0.557888	1.267172	0.451154
8	-0.481649	2.479420	0.634731
6	1.954960	0.946232	0.823015
8	1.402060	-1.867223	0.334279
1	-5.137745	1.218215	-0.411470
1	-5.133234	-1.233881	-0.804774
1	-3.008093	-2.524292	-0.611254
1	-2.996414	2.376392	0.176291
1	2.050487	1.911210	0.321216
1	2.070040	1.160193	1.892341
6	2.689882	-1.437313	0.829515
6	3.050546	-0.029882	0.376571
8	3.205856	-0.096991	-1.033111
1	2.672315	-1.491806	1.922401
1	3.395218	-2.165171	0.434187
1	4.009597	0.239444	0.845514
6	3.830446	1.031682	-1.618989
1	4.810125	1.228379	-1.160271
1	3.974340	0.795655	-2.673257
1	3.216972	1.937128	-1.543615

B3LYP/6-311G(d,p) gas phase computed total energies and Cartesian coordinates of the of the stationary points associated with the HDA reaction between MCDO **1** and ethylene **8**.

8

E(RB3LYP) = -78.6139792323 A.U.

6	-0.663487	0.000000	0.000003
6	0.663487	0.000000	-0.000003
1	-1.234629	-0.922522	-0.000010
1	-1.234629	0.922523	0.000023
1	1.234629	0.922522	0.000010
1	1.234629	-0.922523	-0.000023

TS-4

E(RB3LYP) = -689.081490518 A.U.

6	-2.599633	0.375757	-0.718877
6	-1.219873	0.512281	-0.510552
6	-0.438187	-0.681932	-0.488756
8	-0.990975	-1.800409	-0.621530
6	-2.786834	-1.880873	0.819963
6	-3.391585	-0.637599	0.826075
6	-0.677434	1.799830	-0.083221
6	1.497710	0.742641	0.067764
6	0.994720	-0.536000	-0.201163
8	-1.304255	2.814186	0.084063
8	0.697759	1.854280	0.122461
1	-3.198407	1.277730	-0.665102
1	-2.914306	-0.372500	-1.433385
1	-4.398473	-0.562334	0.428579
1	-3.161270	0.056234	1.626120
1	-3.169625	-2.688789	0.211427
1	-2.002598	-2.141635	1.515754
6	3.224998	-1.442063	0.042669
6	3.712032	-0.155134	0.302131
6	2.855847	0.937478	0.317635
6	1.874760	-1.626642	-0.206636
1	3.902346	-2.287554	0.034152
1	4.768228	-0.004968	0.494735
1	3.210499	1.939743	0.522471
1	1.464246	-2.606851	-0.416897

TS-6

E(RB3LYP) = -689.078566507 A.U.

6	2.399302	0.963868	-0.737055
6	1.105072	0.437149	-0.550914
6	1.019492	-0.979496	-0.463075
8	2.012360	-1.710628	-0.488335
6	3.581932	-0.793060	0.953099
6	3.481491	0.586002	0.831107
6	-0.022162	1.300310	-0.201760
6	-1.329386	-0.823781	-0.069616
8	0.071757	2.518405	-0.085148
1	2.458726	2.047482	-0.753203
1	3.050596	0.446820	-1.429498
1	4.353519	1.109031	0.449833

1	2.923629	1.130687	1.584569
1	4.334308	-1.351385	0.412497
1	3.003295	-1.352453	1.674028
8	-0.195259	-1.583525	-0.226716
6	-1.301619	0.575122	-0.028537
6	-2.527625	-1.523769	0.074287
6	-2.503991	1.268053	0.170578
6	-3.698356	0.583499	0.317016
6	-3.705376	-0.816602	0.267484
1	-2.509085	-2.605787	0.037560
1	-2.456606	2.350011	0.200731
1	-4.624199	1.125702	0.467682
1	-4.637702	-1.357907	0.381715

CA-4
E(RB3LYP) = -689.160119165 A.U.

6	-2.777249	0.541042	-0.002292
6	-1.271065	0.569928	-0.040768
6	-0.527956	-0.572853	-0.035006
8	-1.055261	-1.814080	-0.049296
6	-2.473193	-1.919474	-0.308235
6	-3.261800	-0.847370	0.428220
6	-0.623953	1.870008	-0.044648
6	1.515512	0.746504	-0.000898
6	0.919500	-0.522770	-0.002960
8	-1.190787	2.932749	-0.054927
8	0.773523	1.887169	-0.029947
1	-3.132416	1.314990	0.682059
1	-3.184302	0.804212	-0.985947
1	-4.327281	-0.976688	0.219956
1	-3.120849	-0.979955	1.505485
1	-2.622060	-1.840267	-1.391132
1	-2.738769	-2.927237	0.009212
6	3.119903	-1.526631	0.049963
6	3.698427	-0.251358	0.052405
6	2.903253	0.885073	0.025626
6	1.741526	-1.660756	0.020305
1	3.749731	-2.407853	0.069577
1	4.777018	-0.146371	0.074327
1	3.326235	1.881661	0.025124
1	1.275465	-2.637250	0.014411

CA-6
E(RB3LYP) = -689.147111773 A.U.

6	2.463262	1.282656	-0.016789
6	1.125131	0.587774	-0.044961
6	1.057086	-0.769889	-0.021891
8	2.091999	-1.612619	-0.018676
6	3.386591	-1.022586	-0.295694
6	3.560214	0.307313	0.422369
6	-0.098713	1.372739	-0.055638
6	-1.284978	-0.821472	0.016432
8	-0.104401	2.600143	-0.075093
1	2.398531	2.139262	0.658309
1	2.691018	1.699170	-1.005128
1	4.555765	0.703646	0.204477
1	3.505643	0.136274	1.502119

1	3.469181	-0.898542	-1.380843
1	4.103238	-1.775216	0.029234
8	-0.080629	-1.488071	0.013048
6	-1.347560	0.572273	-0.025074
6	-2.434548	-1.608965	0.058646
6	-2.609242	1.182841	-0.025378
6	-3.761945	0.416792	0.014605
6	-3.671211	-0.981387	0.056860
1	-2.337261	-2.686911	0.090397
1	-2.639220	2.265327	-0.056207
1	-4.734305	0.894953	0.013908
1	-4.572870	-1.582129	0.088582

B3LYP/6-311G(d,p) gas phase computed total energies and Cartesian coordinates of the of the stationary points associated with the more favorable *pseudocyclic* selective reaction paths of the HDA reaction between MCDO **1** and EVE **2**.

EVE **2**
E(RB3LYP) = -232.498721311 A.U.

6	-2.445950	-0.295399	0.023496
6	-1.283477	0.347255	-0.039829
8	-0.086349	-0.296888	-0.026078
1	-2.487763	-1.374153	0.105034
1	-3.372296	0.260306	-0.014835
1	-1.230203	1.431910	-0.121672
6	1.072884	0.538156	0.057906
1	1.056984	1.096639	1.002591
1	1.064764	1.263110	-0.766582
6	2.297110	-0.352424	-0.020704
1	2.308176	-0.905240	-0.962418
1	3.207570	0.249271	0.040882
1	2.300160	-1.072269	0.800413

TS-Et-6on
E(RB3LYP) = -842.987388799 A.U.

6	-3.465589	1.231620	0.688465
6	-3.965404	0.348806	-0.276658
6	-3.170630	-0.669935	-0.783887
6	-1.860927	-0.811957	-0.321697
6	-1.343395	0.064515	0.639957
6	-2.163028	1.085865	1.138714
8	-1.126335	-1.827202	-0.869883
6	0.187491	-2.090300	-0.476833
6	0.727821	-1.278488	0.599539
6	0.043942	-0.117237	1.112983
8	0.594768	0.692079	1.879256
6	2.018247	-1.563221	1.070721
8	0.767741	-2.970405	-1.065154
1	-4.096165	2.019626	1.082905
1	-4.983841	0.455728	-0.632937
1	-3.537353	-1.363380	-1.530359
1	-1.743395	1.742997	1.891242
1	2.426690	-2.535961	0.824079
1	2.270702	-1.163393	2.043912
6	3.432998	-0.593929	0.028916
6	3.029407	0.722135	0.104690
8	2.171762	1.193220	-0.768829
1	3.283023	-1.115086	-0.909259
1	4.302794	-0.874621	0.609602
1	3.313509	1.371540	0.929102
6	1.611384	2.517831	-0.512548
1	1.087724	2.460791	0.444668
1	2.444042	3.223451	-0.438642
6	0.687028	2.858138	-1.660071
1	0.259273	3.850591	-1.498790
1	1.225054	2.863491	-2.610161
1	-0.131603	2.139631	-1.722230

TS-Et-4mn
E(RB3LYP) = -842.987348667 A.U.

6	4.042723	-0.019318	-0.839642
6	3.956733	1.133045	-0.049155
6	2.804390	1.410670	0.673519
6	1.727358	0.525856	0.607005
6	1.796575	-0.632627	-0.173734
6	2.966984	-0.890681	-0.898572
8	0.609073	0.869952	1.326127
6	-0.504649	0.040285	1.375496
6	-0.449737	-1.214302	0.674814
6	0.643831	-1.562338	-0.219575
8	0.624354	-2.550282	-0.953568
6	-1.570034	-2.057261	0.772854
8	-1.473321	0.463527	1.984910
1	4.946496	-0.227445	-1.400014
1	4.795314	1.818361	0.003254
1	2.716309	2.296455	1.290369
1	2.992629	-1.793085	-1.497718
1	-2.194198	-1.952231	1.650087
1	-1.428366	-3.065888	0.400479
6	-2.939517	-1.623349	-0.595124
6	-3.198749	-0.283585	-0.385388
8	-2.442053	0.623936	-0.953194
1	-2.344766	-1.890911	-1.460832
1	-3.718173	-2.319546	-0.308276
1	-3.945495	0.060343	0.326639
6	-2.605663	2.010010	-0.515399
1	-2.361447	2.037304	0.549057
1	-3.653640	2.284763	-0.665681
6	-1.674423	2.867333	-1.342171
1	-1.904296	2.786606	-2.406561
1	-1.783787	3.912454	-1.042254
1	-0.635944	2.573329	-1.182318

CA-Et-4mn
E(RB3LYP) = -843.035527929 A.U.

6	-4.715226	-0.711317	0.012527
6	-4.139395	-1.986649	-0.069354
6	-2.761197	-2.138403	-0.098269
6	-1.961382	-0.997977	-0.042904
6	-2.509413	0.283208	0.037583
6	-3.904974	0.410289	0.064788
8	-0.599227	-1.198032	-0.067372
6	0.213707	-0.125173	-0.024913
6	-0.201389	1.167635	0.036190
6	-1.621839	1.471610	0.081328
8	-2.058937	2.617311	0.138899
6	0.809257	2.284866	0.023616
8	1.479615	-0.541564	-0.059852
1	-5.793462	-0.606107	0.033939
1	-4.772574	-2.865417	-0.111071
1	-2.291739	-3.112106	-0.161354
1	-4.313161	1.411928	0.125881
1	0.884035	2.734105	1.020953
1	0.445313	3.080591	-0.630210
6	2.176198	1.777592	-0.445777

6	2.514504	0.464321	0.231893
8	3.705592	-0.024438	-0.241385
1	2.180512	1.600321	-1.524825
1	2.969846	2.493498	-0.222977
1	2.522351	0.558635	1.328431
6	4.228808	-1.163867	0.462813
1	3.541097	-2.006877	0.350610
1	4.302882	-0.920200	1.531543
6	5.590566	-1.484743	-0.120011
1	6.269462	-0.636859	-0.005871
1	6.024404	-2.349101	0.389159
1	5.505357	-1.716979	-1.183581

CA-Et-6on
E(RB3LYP) = -843.047989159 A.U.

6	2.141417	-3.025195	-0.107912
6	3.441442	-2.507246	-0.057833
6	3.653889	-1.137999	0.007612
6	2.556881	-0.276603	0.021149
6	1.247829	-0.778022	-0.027060
6	1.054198	-2.167062	-0.090631
8	2.811917	1.058361	0.087577
6	1.792751	2.015239	0.088705
6	0.427076	1.526477	0.031544
6	0.168875	0.188893	-0.005672
8	-1.074428	-0.332283	-0.033939
6	-0.679227	2.546580	-0.017027
8	2.120280	3.173362	0.134742
1	1.985962	-4.096083	-0.159547
1	4.292410	-3.178457	-0.070077
1	4.648731	-0.712832	0.047695
1	0.042555	-2.548780	-0.129443
1	-0.386965	3.361755	-0.682598
1	-0.811953	3.005342	0.970128
6	-1.980901	1.896129	-0.493848
6	-2.197200	0.573768	0.215072
8	-3.337055	-0.034422	-0.256176
1	-1.948033	1.693262	-1.567870
1	-2.843550	2.537068	-0.302213
1	-2.234375	0.701582	1.308143
6	-3.784660	-1.175010	0.493015
1	-3.040429	-1.974117	0.424511
1	-3.886508	-0.892495	1.549926
6	-5.114911	-1.617469	-0.083696
1	-5.491774	-2.487599	0.459917
1	-5.852669	-0.815485	-0.010954
1	-5.003860	-1.888192	-1.135763
